# A mathematical framework for yield (*vs.* rate) optimization in constraint-based modeling and applications in metabolic engineering

**DOI:** 10.1016/j.ymben.2018.02.001

**Published:** 2018-05

**Authors:** Steffen Klamt, Stefan Müller, Georg Regensburger, Jürgen Zanghellini

**Affiliations:** a Max Planck Institute for Dynamics of Complex Technical Systems, Magdeburg, Germany; b Faculty of Mathematics, University of Vienna, Austria; c Institute for Algebra, Johannes Kepler University Linz, Austria; d Department of Biotechnology, University of Natural Resources and Life Sciences, Vienna, Austria; e Austrian Centre of Industrial Biotechnology, Vienna, Austria

**Keywords:** ATP, adenosine triphosphate, ECC2, EColiCore2, EFM, elementary flux mode, EFV, elementary flux vector, FBA, flux-balance analysis, gDW, gram dry weight, glc, glucose, GSMM, genome-scale metabolic model, LFP, linear-fractional program, LP, linear program, MCS, minimal cut set, PE, production envelope, PP, phase plane, YS, yield space, Constraint-based modeling, Elementary flux mode, Elementary flux vector, Flux-balance analysis, Linear-fractional program, Metabolic pathway analysis, Production envelope, Productivity, Strain design, Yield space

## Abstract

*Background:* The optimization of metabolic rates (as linear objective functions) represents the methodical core of flux-balance analysis techniques which have become a standard tool for the study of genome-scale metabolic models. Besides (growth and synthesis) rates, metabolic yields are key parameters for the characterization of biochemical transformation processes, especially in the context of biotechnological applications. However, yields are ratios of rates, and hence the optimization of yields (as nonlinear objective functions) *under arbitrary linear constraints* is not possible with current flux-balance analysis techniques. Despite the fundamental importance of yields in constraint-based modeling, a comprehensive mathematical framework for yield optimization is still missing.

*Results:* We present a mathematical theory that allows one to systematically compute and analyze yield-optimal solutions of metabolic models *under arbitrary linear constraints*. In particular, we formulate yield optimization as a linear-fractional program. For practical computations, we transform the linear-fractional yield optimization problem to a (higher-dimensional) linear problem. Its solutions determine the solutions of the original problem and can be used to predict yield-optimal flux distributions in genome-scale metabolic models. For the theoretical analysis, we consider the linear-fractional problem directly. Most importantly, we show that the yield-optimal solution set (like the rate-optimal solution set) is determined by (yield-optimal) elementary flux vectors of the underlying metabolic model. However, yield- and rate-optimal solutions may differ from each other, and hence optimal (biomass or product) yields are not necessarily obtained at solutions with optimal (growth or synthesis) rates. Moreover, we discuss phase planes/production envelopes and yield spaces, in particular, we prove that yield spaces are convex and provide algorithms for their computation. We illustrate our findings by a small example and demonstrate their relevance for metabolic engineering with realistic models of *E. coli*.

*Conclusions:* We develop a comprehensive mathematical framework for yield optimization in metabolic models. Our theory is particularly useful for the study and rational modification of cell factories designed under given yield and/or rate requirements.

## Highlights

• Comprehensive theory of yield analysis and optimization in constraint-based metabolic models is developed.
• Calculation of optimal yields and yield spaces in (genome-scale) metabolic models.
• Differences between rate-optimal and yield-optimal flux distributions are highlighted.
• Design strategies for of yield *vs.* rate optimal cell factories are discussed and illustrated.

## 1 Introduction

Productivity and yield are crucial characteristics of biotechnological production processes based on microbial cell factories (Nielsen and Keasling, 2016, Sanford et al., 2016). Yield is a relative measure of the efficiency of (bio)chemical conversions. In particular, it is the amount of product or biomass formed per amount of substrate consumed. In contrast, productivity measures the speed of product formation, *i.e.*, the amount of product or biomass formed per unit of time. Thereby, one is mainly concerned with productivity quantified by *specific* production rate (*e.g.*, mmol product per gram dry weight and hour) or specific growth rate (with unit per hour).

Yield and productivity are not independent of each other. Moreover, in the case of biomass as the (natural) product, trade-offs between (growth) yield and (growth) rate are believed to shape the evolutionary trajectories of microorganism (Goel et al., 2012). For instance, higher growth yields allow an organism to produce more progenies for the same amount of nutrients, while higher growth rates support faster proliferation, but are often accompanied by reduced biomass yields. The latter growth strategy may be better suited under nutrient excess in order to overgrow any competitors, while the former provides a fitness advantage under nutrient scarcity (Schuster et al., 2008). Another example where the difference between growth yield and growth rate maximization has been discussed intensely in the literature is in the context of respiration *vs.* fermentation (Schuster et al., 2015, Schuster et al., 2011, Simeonidis et al., 2010).

Optimal yields and rates and their trade-offs are frequently studied with the help of mathematical models. Here, evolutionary pressure is often modeled in terms of some kind of optimality. Constraint-based modeling represents one approach to investigate optimality principles based on the underlying metabolic network structure (Bordbar et al., 2014). Flux-balance analysis (FBA) is a particularly prominent constraint-based modeling approach that predicts steady-state flux distributions in genome-scale metabolic models (GSMMs) by assuming an objective function that the cell aims to optimize (Fell and Small, 1986, Orth et al., 2010, Varma and Palsson, 1994, Watson, 1984). Mathematically, FBA is formulated as a linear program (LP) maximizing a single reaction rate or a linear combination of rates. Typically maximizing growth rate is used as a proxy for evolutionary pressure that improves fitness. Other objective functions have been proposed as well (Schuetz et al., 2012, Schuetz et al., 2007, Gianchandani et al., 2008). In this work, we neither address whether (microbial) cells perform optimally at all nor do we claim what rates or yields could be true biological objectives. Instead, we provide computational means to identify yield-optimal flux distributions for any constraint-based metabolic model.

In its mathematical formulation, FBA clearly maximizes rates, however, it has also been used for maximizing yield. In some applications, limiting substrate uptake rates $r_{S}$ are fixed to experimentally measured values. Since biomass yield $Y^{B/S}$ is the ratio of growth rate μ and uptake rate $r_{S}$, maximizing yield is then equivalent to maximizing growth rate (Teusink and Smid, 2006). In other cases, only steady-state and irreversibility constraints are known. Then the optimization of rates *via* FBA leads to infinite values, and only the optimization of product (or biomass) yields $Y^{P/S}=r_{P}/r_{S}$ is meaningful. In a normalization step, the substrate uptake is fixed, *e.g.* $r_{S}=1$, and the (normalized) product formation rate $r_{P}$ is maximized *via* FBA (Schuster et al., 2008, Schuster et al., 2000). For medium-scale networks, this case can also be addressed in the framework of elementary flux mode (EFM) analysis (Schuster and Hilgetag, 1994, Schuster et al., 1999, Schuster et al., 2000, Zanghellini et al., 2013).

For unknown substrate uptake rate and in the presence of other constraints (*e.g.,* lower or upper flux bounds), the situation is different, and the methods described above cannot be used. Here, the nonlinear yield $Y^{P/S}=r_{P}/r_{S}$ has to be maximized explicitly since the substrate uptake rate at the maximum product yield is not known. In particular, rate optimization $\left(r_{P}\to \max\right)$ and yield optimization $\left(r_{P}/r_{S}\to \max\right)$ may then lead to different solutions.

Although (maximal) yields are of central interest in metabolic modeling and engineering, a comprehensive mathematical framework for yield analysis and optimization in the context of constraint-based modeling is still missing. In the present work, we study yield optimization *under arbitrary linear constraints* as a linear-fractional program (LFP), as opposed to rate optimization in FBA which is studied as an LP. For practical computations, we use that a linear-fractional yield optimization problem can be transformed to an equivalent, but higher-dimensional linear problem (which cannot be interpreted as an FBA problem in a straightforward way). By solving this LP and transforming the solutions back to the LFP, we can compute yield-optimal flux distributions in GSMMs.

In our theoretical analysis, we furthermore show that yield-optimal solution sets (like rate-optimal solution sets) can be characterized in terms of EFMs and elementary flux vectors (EFVs) (Klamt et al., 2017). Throughout our study, we emphasize similarities and differences of rate and yield optimization, in particular, in the context of metabolic engineering. We discuss the concepts of phase planes (PPs) and yield spaces (YSs) as important tools for computer-aided strain design and, as another theoretical result, we prove the convexity of YSs.

## 2 Rate and yield optimization

### 2.1 Basic terminology and examples

A metabolic network is represented by its stoichiometric matrix $N\in R^{m\times n}$ containing the net stoichiometric coefficients of *m* internal metabolites in *n* reactions. The vector $r\in R^{n}$ denotes a flux distribution (flux vector) through the network, and its components $r_{i}$ with i∈{1,...,n} are the respective reaction rates or fluxes.

Flux-balance analysis (FBA) identifies particular flux distributions through a metabolic network by optimizing a linear objective function $c^{T}r$ subject to steady-state (2a), capacity and irreversibility (2b), as well as possible additional linear constraints (2c), *e.g.*, for resource allocation (Mori et al., 2016):

(1) $$\max\;c^{T}r$$

subject to

(2a) Nr=0,

(2b) $$r^{\mathrm{lb}}\leq r\leq r^{\mathrm{ub}},$$

(2c) Gr≤h.

The constraints (2b) include irreversibility constraints, where $r_{i}^{\mathrm{lb}}=0$ for irreversible reactions $i\in I_{\mathrm{irr}}$. The additional constraints in (2c) are expressed by a set of *q* linear inequalities represented by a matrix $G\in R^{q\times n}$ and a vector $h\in R^{q}$. The objective function together with the linear constraints form a linear program (LP), which can be solved with standard LP solvers.

A related, but mathematically different type of optimization is the identification of yield-optimal flux vectors. A yield is the *ratio* of two fluxes or, more generally, the ratio of linear combinations of fluxes,

(3) $$Y(r)=\frac{c^{T}r}{d^{T}r}.$$

Usually, the numerator contains a sum of (weighted) product fluxes, whereas the denominator contains a sum of (weighted) substrate uptake fluxes and is assumed to be positive. Thereby, the directions of substrate uptake reactions are fixed, and the corresponding signs of fluxes and coefficients match. That is, for uptake reaction *i*, either $r_{i}\geq 0$ and $d_{i}\geq 0$ or $r_{i}\leq 0$ and $d_{i}\leq 0$.

Yield optimization poses the following problem:

(4) max Y(r)

subject to the same constraints (2a), (2b), (2c) as for FBA and the additional assumption $d^{T}r>0$. Since the objective function is a fraction of two linear functions, this kind of optimization problem is called a linear-fractional program (LFP) (Boyd and Vandenberghe, 2004, Frenk and Schaible, 2005, Maranas and Zomorrodi, 2016). We note that, in a general LFP, the objective function has the form $\left(c^{T}r+p)/(d^{T}r+q\right)$. In the case of yield optimization, we have p=q=0 which simplifies the mathematical treatment.

**Fig. 1:**
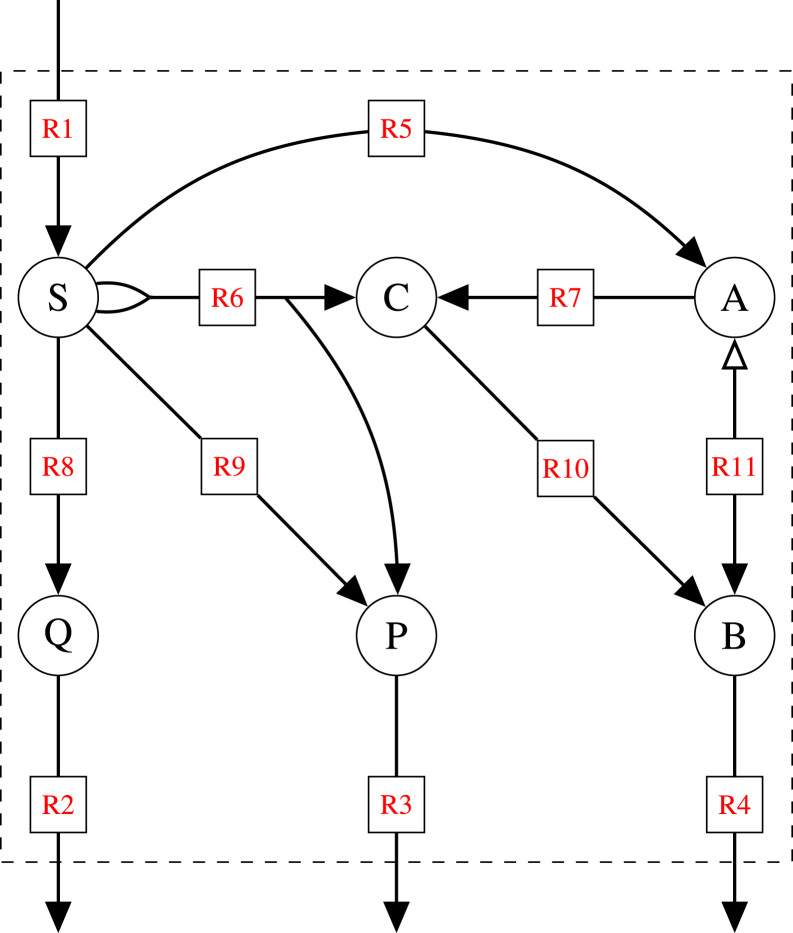
Example network with six (internal) metabolites (A, B, C, P, Q, S), ten irreversible reactions (R1–R10), and one reversible reaction (R11), where the open arrow indicates the backward direction. Every (incoming or outgoing) edge of a node represents a stoichiometric coefficient of one. Thus, reaction R6 uses two molecules of S and produces one C and one P.

**Fig. 2:**
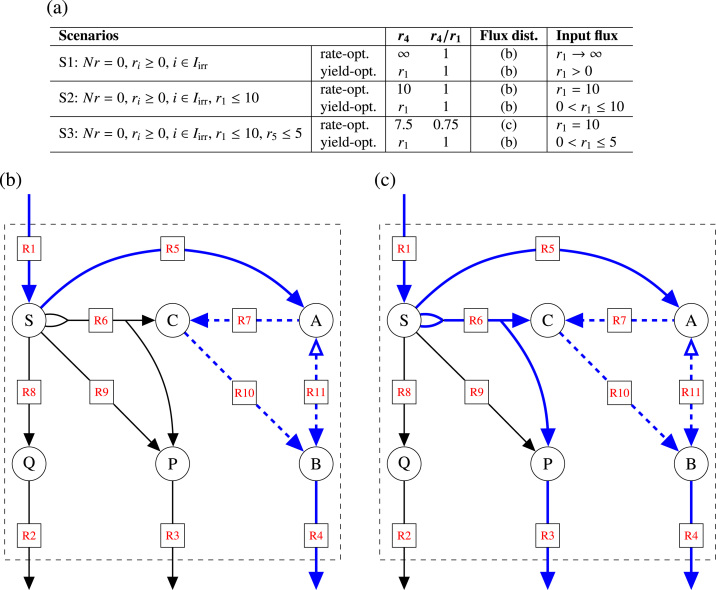
Rate- and yield-optimal flux distributions for scenarios S1–S3 of the example network in Fig. 1. Panel (a) lists the main characteristics of the three scenarios together with the respective rate- and yield-optimal flux distributions (depending on the input flux $r_{1}$). The contributing reactions are illustrated as blue lines in panels (b) and (c). Full lines indicate fluxes depending only on $r_{1}$, while dashed lines indicate fluxes depending also on $r_{11}$.

In the following, we will use the example network in Fig. 1 to illustrate rate- and yield-optimal flux distributions in three different scenarios. With reaction R1 as S(ubstrate) uptake, we are particularly interested in the synthesis of B(iomass or a biomass component) *via* reaction R4. Thereby, P and Q (with excretion reactions R3 and R2) represent two byproducts. In each of the following three scenarios, we are interested in the maximal biomass synthesis rate $r_{4}$ and the maximal biomass yield $Y^{B/S}=r_{4}/r_{1}$. The main characteristics of these scenarios are summarized in Fig. 2(a).

*Scenario S1*. First, we only consider the steady-state and irreversibility constraints. Clearly, maximization of the biomass synthesis rate $r_{4}$ is then meaningless since the resulting LP is unbounded, leading to an infinite rate $r_{4}$. In contrast, the optimal B(iomass) yield is one, irrespective of the amount of substrate taken up. See Fig. 2(a) and (b).

*Scenario S2*. Next, we use the (standard) condition of Scenario S1 and restrict the substrate uptake rate by $r_{1}\leq 10$. Now, the maximum rate for biomass synthesis is ten, whereas the maximum biomass yield is still one. In this case, the maximum biomass rate is just given by the substrate uptake rate multiplied by the maximum biomass yield. Note that for $r_{1}$ infinitely many solutions exist that result in yield-optimality $\left(0<r_{1}\leq 10\right)$, whereas only one solution results in rate-optimality $\left(r_{1}=10\right)$. See Fig. 2(a) and (b).

*Scenario S3*. Finally, we also set a capacity constraint for reaction R5: $r_{5}\leq 5$. Apparently, the maximum rate for biomass synthesis is now 7.5, whereas the maximum biomass yield is still one. However, the maximum yield can only be reached for substrate uptake rates $r_{1}\leq 5$, whereas the maximum rate occurs at $r_{1}=10$. As a consequence, the rate-optimal solution is not yield-optimal. See Figs. 2(a)–(c).

*Remark*. Note that, in some special cases, the maximum yield is reached only for infinitely large fluxes (one says “it is not attained”). If we take scenario S1 and add a nonzero lower bound for reaction R2, *e.g.* $r_{2}\geq 1$, then the maximum B(iomass) yield of one is approached for very large substrate uptake flux $r_{1}$ (but will never be attained).

Numerous LPs, given by Eq. (1) subject to the constraints (2a), (2b), (2c), have been solved in the context of FBA to find rate-optimal solutions, *e.g.*, growth-rate-optimal phenotypes or flux distributions with optimal product synthesis rates. Optimal yields are obviously also of high interest in the context of metabolic engineering. However, surprisingly, with only one exception (Burgard et al., 2004) (in the context of flux coupling analysis, see Section 2.3), we are not aware of a single constraint-based modeling study that solved the LFP given by (4) subject to (2a), (2b), (2c) to optimize yield in a metabolic network. Instead, yield-optimal solutions have often been identified either *via* elementary flux modes (EFMs) (applicable only in smaller networks, see below) or by fixing the substrate uptake rate and then maximizing product synthesis rate *via* a standard LP (Schuster et al., 2008, Teusink and Smid, 2006). However, these methods cannot be used in the general case of arbitrary constraints. For example, fixing the substrate uptake rate in scenario S3 to its maximum value $r_{1}=10$ and then maximizing the biomass synthesis rate will deliver a flux vector that is not biomass-yield optimal for scenario S3.

In the following, we give a mathematical definition of yield optimization problems in the context of constraint-based modeling and discuss important properties of the resulting LFPs. Further, we show how they can be written as LPs, which allows the use of efficient algorithms even for genome-scale metabolic models (GSMMs). In a subsequent section, we characterize rate- as well as yield-optimal solution sets in terms of rates and yields of (optimal) generators of the flux polyhedron.

### 2.2 Mathematical treatment

We define rate optimization (as an LP) and yield optimization (as an LFP). Most importantly, we rewrite yield optimization as an LP.

The constraints (2a), (2b), (2c) define the flux polyhedron

(5) $$P=\{x\in R^{n}\;|\;\mathrm{Ax}\leq b\},$$

where

(6) $$A=\left(\begin{array}{c} N\\ -N\\ I\\ -I\\ G \end{array}\right),\;b=\left(\begin{array}{c} 0\\ 0\\ r^{\mathrm{ub}}\\ -r^{\mathrm{lb}}\\ h \end{array}\right),$$

and $I\in R^{n\times n}$ is the identity matrix. As standard in convex optimization, we use the variable $x\in R^{n}$ for the (flux) vector $r\in R^{n}$.

#### 2.2.1. Definitions

Given a vector $c\in R^{n}$, we define the linear objective function $l:R^{n}\to R$,

(7) $$l(x)=c^{T}x$$

and study *rate optimization* as the LP

(8) $$\underset{x\in P}{\max\;}l(x).$$

Given $c,d\in R^{n}$, we define the yield Y:D→R as the rational function

(9) $$Y(x)=\frac{c^{T}x}{d^{T}x}$$

on the set

(10) $$D=\{x\in R^{n}\;|\;d^{T}x>0\}.$$

That is, we require a positive denominator. We study *yield optimization* as the LFP

(11) $$\underset{x\in P_{>}}{\max}Y(x)$$

with

(12) $$P_{>}=P\cap D=\{x\in P\;|\;d^{T}x>0\}.$$

We note that, given rate- and yield-optimal solutions, $x^{*r}$ and $x^{*y}$, respectively, the inequalities $c^{T}x^{*r}\geq c^{T}x^{*y}>0$ and $Y(x^{*y})\geq Y(x^{*r})>0$ imply $d^{T}x^{*r}\geq d^{T}x^{*y}$. In biological terms, the substrate uptake in yield-optimal states is never larger than the substrate uptake in rate-optimal states.

#### 2.2.2. Yield optimization as an LP

In general, an LFP is equivalent to an LP (Boyd and Vandenberghe, 2004, Frenk and Schaible, 2005, Maranas and Zomorrodi, 2016). Let P={x∣Ax≤b} be a polyhedron (with recession cone $\left\{x\in R^{n}\;|\;\mathrm{Ax}\leq 0\right\}$). The above LFP

$$\underset{x\in P_{>}}{\max}\;\frac{c^{T}x}{d^{T}x}$$

with

$$P_{>}=\{x\in P\;|\;d^{T}x>0\}$$

is equivalent to the LP

(13) $$\underset{(x\prime ,t)\in P\prime }{\max}\;c^{T}x\prime$$

with the auxiliary polyhedron

(14) $$P\prime =\{(x\prime ,t)\;|\;\mathrm{Ax}\prime \leq \mathrm{tb},\;d^{T}x\prime =1,\;t\geq 0\}$$

in the following sense: The LFP is feasible if and only if the LP is feasible, and both optimization problems have the same optimal value. In detail, if $x\in P_{>}$ is feasible in the LFP, then (x′,t)∈P′ with

$$x\prime =\frac{x}{d^{T}x}\;\mathrm{and}\;t=\frac{1}{d^{T}x}>0$$

is feasible in the LP with the same objective value.

Conversely, if (x′,t)∈P′ with t>0 is feasible in the LP, then

$$x=\frac{x\prime }{t}\in P_{>}$$

is feasible in the LFP with the same objective value.

Finally, if t=0, then (x′,0)∈P′ corresponds to an element of the recession cone of P since Ax′≤0 (and $d^{T}x\prime =1$). In those cases, the objective value of the LFP approaches the objective value of the LP in the limit: for arbitrary, but fixed $x_{0}\in P_{>}$, we have

$$\{x_{0}+\lambda x\prime \;|\;\lambda \geq 0\}\subseteq P_{>}$$

and

$$\underset{\lambda \to \infty }{\lim}\;\frac{c^{T}(x_{0}+\lambda x\prime )}{d^{T}(x_{0}+\lambda x\prime )}=\frac{c^{T}x\prime }{d^{T}x\prime }=c^{T}x\prime .$$

If the LP has an optimal solution $\left(x^{*},0\right)$, two cases can occur. If there exists another optimal solution $\left(\overset{˜}{x},\overset{˜}{t}\right)$ with $\overset{˜}{t}>0$, then the optimal yield is attained at the corresponding element of $P_{>}$. Otherwise, the optimal yield is reached only in the limit (as already illustrated in the remark in Section 2.1). In practice, one is interested in optimal yields attained at finite fluxes and one can proceed as follows. If the LP solver returns an optimal solution $\left(x^{*},0\right)$, one first determines the feasible range of *t*, by maximizing *t* in the auxiliary polyhedron (14). Then one chooses a feasible $\overset{˜}{t}>0$ close to zero (but numerically unproblematic) and determines an optimal solution $\left(\overset{˜}{x},\overset{˜}{t}\right)$ of (13). If $c^{T}\overset{˜}{x}=c^{T}x^{*}$, then the optimal yield is attained at finite fluxes.

We note that the equivalence of the LP and the LFP also implies that the LP is unbounded if and only if the original LFP is unbounded. Such cases arise, for example, if alternative substrates can be used to synthesize the product, but are not accounted for in the denominator of the yield function (9).

### 2.3 Flux coupling analysis

Flux coupling analysis is a method to detect functionally related reactions (Burgard et al., 2004, Maranas and Zomorrodi, 2016). The fluxes $r_{i}$ and $r_{j}$ of any two reactions *i* and *j* can be either fully, partially, directionally, or not at all coupled. These features can be detected by maximizing and minimizing the flux ratio $r_{i}/r_{j}$ over the set of feasible flux vectors. If $\max\;(r_{i}/r_{j})=\min\;(r_{i}/r_{j})$, then the two fluxes are fully coupled, *i.e.*, one flux is a multiple of the other. If both ratios are not equal, but nonzero and finite, then the reactions are partially coupled. This means that, if any of the two fluxes is nonzero, then the other one is nonzero, too. Finally, if the activity of one reaction implies the activity of the other but not *vice versa*, then the reactions are directionally coupled. This is the case when one of the ratios is finite and the other is zero. For example, in the network in Fig. 1, the fluxes $r_{2}$ and $r_{8}$ are fully coupled, whereas $r_{6}$ and $r_{1}$ are directionally coupled, and no pair of reactions is partially coupled.

Maximizing and minimizing a flux ratio is a special case of optimizing a rational function as in (4). In fact, Burgard et al. (2004) used an LFP for flux coupling analysis, but they did not extend their work to the analysis of yields.

## 3 Rate-optimal and yield-optimal solution sets

### 3.1 Basic terminology and examples

In this section we show how optimal rates and yields and the corresponding rate- and yield-optimal solution sets can be analyzed by means of *generating* vectors.

If the constraints (2a), (2b), (2c) for the objective functions (1), (4) contain only the steady-state and irreversibility constraints, then the set of feasible flux vectors forms a polyhedral cone, the *flux cone*. A well-known and particularly useful generating set of the flux cone is given by the set of *elementary flux modes (EFMs)*, which are non-decomposable (support-minimal) flux vectors (Schuster and Hilgetag, 1994, Schuster et al., 1999, Schuster et al., 2000, Zanghellini et al., 2013). Every feasible steady-state flux distribution is a conical (nonnegative linear) combination of EFMs, and it is well-known that EFMs can be used to identify yield-optimal pathways. (For examples, see below.)

Rate maximization on the (unbounded) flux cone usually yields an unbounded maximum and is thus meaningless. Therefore, FBA usually involves constraints in (2b), (2c) that go beyond steady state and irreversibility (*e.g.*, maximal substrate uptake rates or resource allocation constraints). This usually bounds the feasible solutions, or at least the value of the objective function, see scenarios S2 and S3 in Fig. 2(a). The resulting *flux polyhedron* cannot be analyzed with EFMs, but with the more general approach of *elementary flux vectors (EFVs)* (Urbanczik, 2007, Klamt et al., 2017, Müller and Regensburger, 2016). EFVs are a particularly useful generating set of a flux polyhedron and generalize EFMs, since EFVs and EFMs coincide in the case of a flux cone. Only recently it has been realized that EFVs can indeed be used to characterize rate- *and* yield-optimal solution sets (of linear programs (LPs) and linear-fractional programs (LFPs), respectively (Klamt et al., 2017)).

For the three scenarios S1–S3 of the example network in Fig. 1, the corresponding EFMs and EFVs are listed in Table 1. Here, we illustrate how they can be used to characterize the sets of rate- and yield-optimal flux vectors.

**Table 1:** List of generators (EFMs and EFVs) for scenarios S1 to S6 in the example network in Fig. 1. Scenarios are characterized by an increasing number of constraints (indicated by “*”). Membership of generators in different scenarios is indicated by “+”. Generators are characterized by their B(iomass) yield $Y^{B/S}$ and P(roduct) yield $Y^{P/S}$. Some generators are not bounded (indicated in the column “Bounded”), and normalized rates are then listed.

*Scenario S1*. The standard condition involves only the steady-state and irreversibility constraints of the network. These constraints form the flux cone which is generated by the six EFMs listed in Table 1. Note that the first five EFMs represent pathways from S(ubstrate) to products (B, P, Q), whereas EFM6 is an internal cycle involving the reactions R7, R10, and R11. (Such a cycle is thermodynamically infeasible, but we keep it for illustrating the concept.) Except for the cycle, all EFMs have a well-defined B(iomass) yield $Y^{B/S}=r_{4}/r_{1}$, and the maximum yield of $Y^{B/S}=1$ is reached by EFM4 and EFM5. As will be detailed below, the set of yield-optimal flux distributions is given by all possible conical sums of EFM4, EFM5 (having maximum yield) and the cycle EFM6 (with undefined yield). Thereby, EFM4 and/or EFM5 must contribute to the sum. All these flux distributions, indicated in Fig. 2(b), have maximum yield. Again, note that rate-optimal fluxes are unbounded in flux cones.

*Scenario S2.* The substrate uptake flux is constrained by an upper bound, and the set of feasible solutions changes from a flux cone to a flux polyhedron. Since EFMs are not defined for general (flux) polyhedra, we determine the EFVs. Indeed, the flux polyhedron is generated by seven EFVs (Table 1). Six of them correspond to the EFMs of S1: EFV7-EFV11 correspond to EFM1-EFM5 but are now scaled to the maximal substrate uptake rate, whereas EFV22 is identical to the cycle EFM6 and remains unscaled. Finally, EFV23 is the zero vector (which is always an EFV if it is contained in the flux polyhedron). Now we can characterize solutions with maximum biomass production rate and maximum biomass yield, respectively. Similarly to scenario S1, the yield-optimal solution set is given by all possible *convex* sums of EFV10 and EFV11 (with a maximum yield of $Y^{B/S}=1$), and the zero vector EFV23 plus some nonnegative multiple of the cycle EFV22. Although EFV22 and EFV23 have undefined yield, they do not affect the overall (maximum) yield as long as at least one EFV with maximum yield (EFV10 and/or EFV11) contributes to the sum. These flux distributions are again illustrated in Fig. 2(b). In contrast, the set of rate-optimal flux vectors (with a maximum rate of $r_{4}=10$) is given by all convex sums of (only) EFV10 and EFV11 plus some nonnegative multiple of the cycle EFV22.

*Scenario S3*. Finally, with the additional flux bound $r_{5}\leq 5$, we find that the B(iomass) yield-optimal solutions are now described by EFV18 and EFV19, which correspond to EFV10 and EFV11 in scenario S2, but are now scaled to the additional flux bound. The yield-optimal solution set is given by all convex sums of EFV18 and EFV19 (having maximum yield $Y^{B/S}=1$) and the zero vector EFV23 plus some nonnegative multiple of the cycle EFV22 (again, with some minimum contribution of EFV18 and/or EFV19). In contrast, the set of rate-optimal solutions (with a maximum rate of $r_{4}=7.5$) is given by all convex sums of the (newly arising) EFV16 and EFV17 plus some nonnegative multiple of the cycle EFV22, see Fig. 2(c).

### 3.2 Rate-optimal solution sets

Recently, a general guideline has been given how to use EFVs (or EFMs in the special case of flux cones) to describe rate- and yield-optimal solution sets (Klamt et al., 2017). For the case of yield optimization, a detailed mathematical treatment will be given in 3.3, 3.4. Before that, we summarize the results for the simpler case of rate optimization,

$$\underset{x\in P}{\max\;}c^{T}x.$$

By Minkowski's Theorem, the flux polyhedron $P=\{x\in R^{n}\;|\;\mathrm{Ax}\leq b\}$ is the sum of a polytope (a bounded polyhedron) and a finitely generated cone, the recession cone $\left\{x\in R^{n}\;|\;\mathrm{Ax}\leq 0\right\}$. The linear objective $c^{T}x$ is bounded on P if and only if it vanishes on the recession cone. In this case, we call the LP bounded.

Most importantly, if the LP is bounded, then every optimal solution is a convex sum of optimal generators of the polytope plus a conical sum of generators of the recession cone – and *vice versa*. Hence, the optimal solution set is a (sub)polyhedron. It is the sum of the optimal (sub)polytope and the recession cone.

Any set of generators (of P) can be used to characterize the rate-optimal solution set, for example, the set of minimal generators (as used in (Kelk et al., 2012)) or the set of EFVs. In fact, EFVs have several valuable properties for characterizing (optimal) solution sets (Klamt et al., 2017), and we suggest to use EFVs as in the scenarios S2 and S3 above.

### 3.3 Mathematical properties of yield optimization

In order to characterize optimal solution sets of the yield optimization problem, we require a number of auxiliary results. For detailed statements and proofs, see the Appendix.

Property 1 *In the definition of the yield Y*, *the numerator* $c^{T}x$ *and the denominator* $d^{T}x$ *do not contain constant terms and hence*

(15) Y(λx)=Y(x)

*for* x∈D *and* λ>0. *That is*, *Y is constant on the ray* {λx∣λ>0}.

Property 2 *The yield Y is not a linear function, not even convex (or concave). Still, for* x,y∈D *and* λ∈[0,1], *the yield of the convex combination*

z=(1−λ)x+λy

*is given by the convex combination of yields*

Y(z)=(1−λ′)Y(x)+λ′Y(y)

*with a unique* λ′∈[0,1]. *See* Proposition 1 *in the* Appendix.

Property 3 *The domain* $P_{>}$ *is contained in the polyhedron*

(16) $$P_{\geq }=\{x\in P\;|\;d^{T}x\geq 0\}.$$

*Clearly*,

$$P_{\geq }=P_{>}\cup P_{=},$$

*where*

(17) $$P_{=}=\{x\in P\;|\;d^{T}x=0\}.$$

*In fact,* $P_{\geq }$ *is the smallest polyhedron containing* $P_{>}$.

Again, by Minkowski's Theorem, the polyhedron $P_{\geq }$ is the sum of a polytope and a finitely generated cone (the recession cone of $P_{\geq }$). Explicitly, let $v^{i}\in P_{\geq }$ (i∈I) and $u^{j}\in \mathrm{rec}(P_{\geq })$ (j∈J) be generators of the polytope and the recession cone, respectively (*I* and *J* are finite index sets of these generators). Then, every $x\in P_{\geq }$ (in particular, every $x\in P_{>}$) can be written as

(18) $$x=\underset{i\in I}{\sum }\alpha_{i}v^{i}+\underset{j\in J}{\sum }\beta_{j}u^{j}$$

with $\alpha_{i},\beta_{j}\geq 0$ and $\sum_{i\in I}\alpha_{i}=1$. Again, any set of generators (of $P_{\geq }$) can be used and we suggest to deploy the set of EFVs (Urbanczik and Wagner, 2005, Urbanczik, 2007, Klamt et al., 2017, Müller and Regensburger, 2016).

In our main result below, we characterize optimal solutions of the yield optimization problem in terms of generators (EFVs) of $P_{\geq }$.

Property 4 *A vector* $x\in P_{=}$ (*with* $d^{T}x=0$) *may have unbounded yield* (*if* $c^{T}x\neq 0$) *or undefined yield* (*if* $c^{T}x=0$). *That is*,

$$P_{=}=P_{\pm /0}\cup P_{0/0},$$

*where*

(19) $$\begin{array}{ccc} P_{\pm /0} & = & \{x\in P_{=}\;|\;c^{T}x\neq 0\}\text{,}\\ P_{0/0} & = & \{x\in P_{=}\;|\;c^{T}x=0\}. \end{array}$$

The definition of the yield directly implies the following two results:

On the one hand, if there is a vector in $P_{\geq }$ with unbounded yield (that is, a vector in $P_{\pm /0}$), then *Y* is unbounded on $P_{>}$. See Lemma 1 in the Appendix.

On the other hand, the addition of a vector in $P_{\geq }$ with undefined yield (that is, a vector in $P_{0/0}$) to a vector in $P_{>}$ does not change the yield. See Lemma 2 in the Appendix.

As a consequence, if *Y* is bounded on $P_{>}$, then generators of $P_{\geq }$ cannot have unbounded yield, but may have undefined yield.

Property 5 *Most importantly*, *if Y is bounded, then the yield of a vector in* $P_{>}$ *is a convex sum of the yields of generators of* $P_{\geq }$ *with defined yield. See* Lemma 3 *in the* Appendix*. The corresponding index sets are given by*

(20) $$\begin{array}{ccc} I^{d} & = & \{i\in I\;|\;v^{i}\in P_{>}\}\text{,}\\ J^{d} & = & \{j\in J\;|\;u^{j}\in P_{>}\}. \end{array}$$

### 3.4 Yield-optimal solution sets

Now we are in a position to characterize optimal solutions of the yield optimization problem in terms of generators of $P_{\geq }$.

First, we note that the maximum yield need not be attained, that is, the maximum is only approached in the limit (as already illustrated in the remark in Section 2.1). The following theorem determines when this is the case.

Theorem 1 *Let the yield Y be bounded on the nonempty* domain $P_{>}$. *Then the maximum is not attained if and only if*

$$I^{d}=I\;\mathrm{and}\;\underset{i\in I^{d}}{\max\;}Y(v^{i})<\underset{j\in J^{d}}{\max\;}Y(u^{j}).$$

*If the maximum is not attained*, *let* $Y^{*}$ *be the supremum. Then* $Y(x)\to Y^{*}$ *for* $x=v+\beta_{j}u^{j}\in P_{>}$ *with* $v\in P_{>}$, $Y(u^{j})=Y^{*}$, *and* $\beta_{j}\to \infty$.

Proof See the Appendix. ▫

If the maximum yield is attained, then the following theorem states that every optimal solution is a convex/conical sum of generators with maximum or undefined yield – and *vice versa*. Thereby, at least one generator with maximum yield contributes to the sum. Hence, the closure of the optimal solution set is a (sub)polyhedron.

Theorem 2 *Let the yield Y be bounded on the* domain $P_{>}$ *and the maximum* $Y^{*}$ *be attained. Then* $x^{*}\in P_{>}$ *is an optimal solution if and only if*

$$x^{*}=\underset{i\in I^{*}}{\sum }\alpha_{i}^{*}v^{i}+\underset{i\in I^{u}}{\sum }\alpha_{i}v^{i}+\underset{j\in J^{*}}{\sum }\beta_{j}^{*}u^{j}+\underset{j\in J^{u}}{\sum }\beta_{j}u^{j},$$

*where*

$$\begin{array}{ccc} I^{*} & = & \{i\in I\;|\;Y(v^{i})=Y^{*}\}\text{,}\\ I^{u} & = & \{i\in I\;\;|\;\;v^{i}\in P_{0/0}\}\text{,}\\ J^{*} & = & \{j\in J\;\;|\;\;Y(u^{j})=Y^{*}\}\text{,}\\ J^{u} & = & \{j\in J\;\;|\;\;u^{j}\in P_{0/0}\}, \end{array}$$

*and* $\alpha_{i}^{*},\alpha_{i},\beta_{j}^{*},\beta_{j}\geq 0$ *with*

$$\underset{i\in I^{*}}{\sum }\alpha_{i}^{*}+\underset{i\in I^{u}}{\sum }\alpha_{i}=1\;\mathrm{and}\;\underset{i\in I^{*}}{\sum }\alpha_{i}^{*}+\underset{j\in J^{*}}{\sum }\beta_{j}^{*}>0.$$

Proof See the Appendix. ▫

With the theoretical results obtained above, we can characterize the yield-optimal solution set in terms of generators of $P_{\geq }$, in particular, in terms of EFVs (see Fig. S1). For realistic applications, we assume that the denominator of the yield function is nonnegative (that is, the flux polyhedron P coincides with $P_{\geq }$) and that the yield is bounded (on the nonempty domain $P_{>}$). The set of EFVs of P consists of “bounded” EFVs *v* of the polytope associated to P and “unbounded” EFVs *u* of the recession cone of P.

Case 1: If the flux polyhedron is the flux cone (given by steady-state and irreversibility constraints), then no “bounded” EFV *v* exists, and the (sub)cone of yield-optimal solutions is the set of conical sums of the EFVs *u* (here coinciding with the set of EFMs) with maximum or undefined yield. Thereby, at least one EFM with maximum yield contributes to the sum.

Case 2: If the flux polyhedron is a polytope (the recession cone is empty), then no EFV *u* exists, and the (sub)polytope of yield-optimal solutions is the set of convex sums of EFVs *v* with maximum or undefined yield. (Note that the zero vector is an EFV with undefined yield if it is contained in the flux polyhedron). Again, at least one EFV with maximum yield contributes to the sum.

Case 3a: If the flux polyhedron is a general polyhedron (generated by “bounded” and “unbounded” EFVs) and at least one of the EFVs *v* has maximum or undefined yield, then the maximum yield is attained, and the (sub)polyhedron of yield-optimal solutions is the set of convex sums of EFVs *v* plus conical sums of EFVs *u* with maximum or undefined yield. Again, at least one EFV (*u* or *v*) with maximum yield contributes to the sum.

Case 3b: If the flux polyhedron is a general polyhedron (as in case 3a) and all EFVs *v* have defined, but not maximum yield, then the maximum yield is *not* attained and only approached in the limit, in particular, for an “infinite” contribution of EFVs *u* with maximum yield.

In Section 3.1, we already applied our theoretical results to characterize yield-optimal solution sets by EFMs (for the flux cone in scenario S1) and EFVs (for the flux polyhedra in scenarios S2 and S3).

## 4 Phase planes and yield spaces

### 4.1 Basic terminology and examples

So far we have analyzed the optimization of a single rate or yield. Now we study (phenotypic) phase planes (PPs) and yield spaces (YSs) which have become an important tool in constraint-based modeling for metabolic networks. A PP is a projection of the flux polyhedron on two (or three) selected fluxes, that is, on a two-dimensional plane or on a three-dimensional space. Similarly, a YS is a map from the flux polyhedron to two (or three) selected yields. Thereby, we assume that the denominator $d^{T}x$ in Eq. (3) is identical for all yields, while the numerators $c^{T}x$ differ, *i.e.*, we consider the same substrate(s), but different products. Note that a YS is not a projection, since yields are nonlinear functions of the flux vectors, see Eq. (3). PPs and YSs allow to analyze dependencies between selected fluxes and yields, respectively. This is particularly useful in the context of metabolic engineering and biotechnological applications (see 5, 6). Growth rate and synthesis rate of a target product are frequently chosen for projection; in this case, the resulting PP is often called production envelope (PE) or trade-off plot (Maranas and Zomorrodi, 2016, Machado and Herrgard, 2015). Likewise, biomass yield and product yield are often chosen for YS analysis.

Again, we use the example network in Fig. 1 to illustrate PPs and YSs in the three scenarios S1–S3. As before, R1 represents S(ubstrate) uptake and R4 (B)iomass synthesis. In addition, we now consider the product P (excreted by R3) with synthesis rate $r_{3}$ and yield $Y^{P/S}=r_{3}/r_{1}$. The product Q is still considered undesired.

**Fig. 3:**
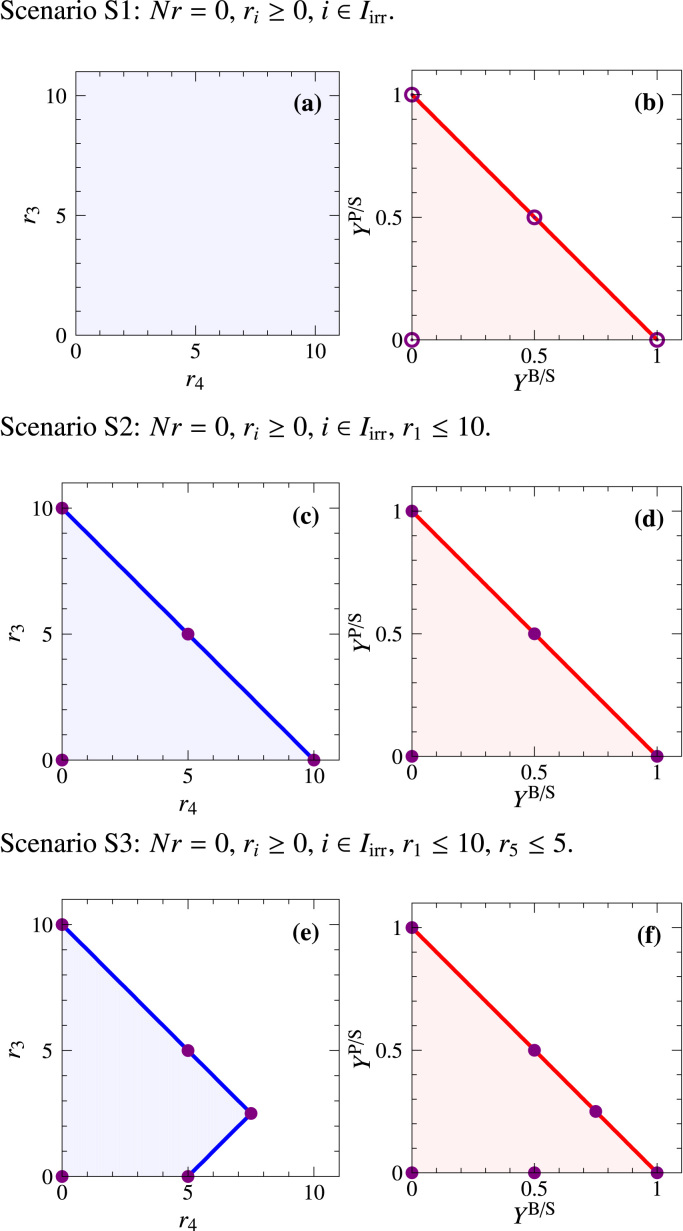
PPs (blue) and YSs (red) for scenarios S1–S3 of the example network in Fig. 1. Open and solid circles correspond to EFMs and EFVs, respectively (*cf.*Table 1). Note that some circles correspond to more than one EFM or EFV.

*Scenario S1*. Since the flux cone is unbounded, the PP is unbounded as well, see Fig. 3(a). In contrast, the YS is bounded, since biomass and product synthesis rates are normalized by the substrate uptake rate, see Fig. 3(b). Both, maximal product yield and maximal biomass yield are one. The triangle shape of the YS indicates the trade-off between biomass and product yields due to mass conservation: the more product is formed, the less biomass can be made from the substrate. However, other shapes of the YS do exist, as will be shown below. Note that every flux distribution of the example network is mapped to exactly one point in the YS. Conversely, every point in the YS corresponds to (possibly infinitely many) flux vectors exhibiting the respective biomass and product yields. For example, all flux distributions indicated in Fig. 2(b) map to the point (1,0) in the YS.

**Fig. 4:**
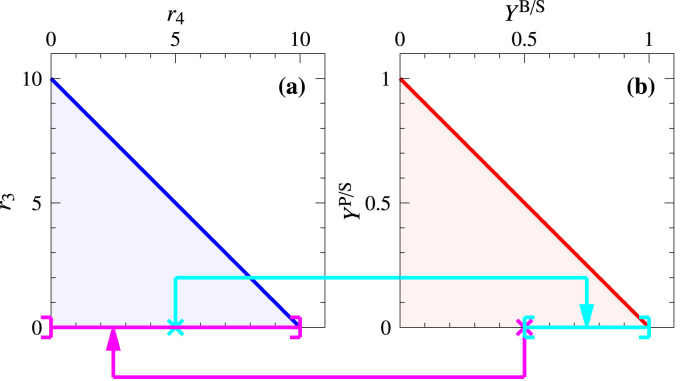
Relationships between PP (blue) and YS (red) of scenario S2. Cyan lines indicate the mapping of the point (5,0) from the PP to the YS. Magenta lines indicate the mapping of the point (0.5,0) from the YS to the PP. Brackets indicate (half-)open and closed intervals, respectively.

*Scenario S2*. Limiting the substrate uptake rate by $r_{1}\leq 10$ also bounds $r_{4}$ and $r_{3}$ and hence the PP, see Fig. 3(c). However, the YS remains the same as in scenario S1, see Fig. 3(d). Note that, up to scaling, the PP and the YS are identical. Still, points (and line segments) of the PP and the YS are not in one-to-one correspondence. For example, the point (5,0) in the PP is the projection of all flux distributions having a biomass synthesis rate $r_{4}=5$ and a product formation rate $r_{3}=0$. Out of those, all flux vectors of the form $r={\left(5,0,0,5,5,0,\lambda ,0,0,\lambda ,5-\lambda \right)}^{T}$ with λ≥0 have a substrate uptake rate $r_{1}=5$ and hence convert all substrate to biomass. That is, their biomass yield is one, and they are mapped to the point (1,0) in the YS, see Fig. 4. However, there are other flux vectors with the same projection (5,0) in the PP: in those solutions, the substrate is taken up with $r_{1}=5+\delta$ (0≤δ≤5) and B is synthesized with $r_{4}=5$ and Q with $r_{2}=\delta$. Therefore, all flux distributions projected to (5,0) in the PP are mapped to the (closed) line segment between (0.5,0) and (1,0) in the YS, see Fig. 4 (full lines). Conversely, flux vectors mapped to (0.5,0) in the YS are projected on the (half-open) line segment between (0,0) and (10,0) in the PP, see Fig. 4 (dashed lines).

Note that flux vectors with undefined yield (*e.g.*, the zero vector or the internal cycle) have no corresponding point in the YS.

*Scenario S3*. The additional capacity constraint $r_{5}\leq 5$ leads to different shapes of the PP and the YS, see Fig. 3(e) and (f). While the YS still forms a triangle (with a maximum biomass yield of 1), the PP becomes a quadrangle (with the maximal biomass synthesis rate dropping from 10 to 7.5). The decrease is caused by the limitation of reaction R5 and the resulting use of reactions R6 and R3 (product formation and excretion). As discussed earlier, rate- and yield-optimal flux distributions differ in this scenario. In particular, optimal biomass yields occur at suboptimal biomass synthesis rates, since all yield-optimal solutions have substrate uptake rate $r_{4}=r_{1}$ with $0<r_{1}\leq 5$. These flux distributions are projected to the line segment between (0,0) and (0,5) in the PP. However, note that every point on the *x*-axis of the PP corresponds to many flux vectors, including those with suboptimal yield.

In the following we give a precise mathematical formulation of YSs and PPs and discuss their properties.

### 4.2 Mathematical treatment

In constraint-based modeling, one often considers two fluxes $x_{i}$ and $x_{j}$ (that is, two components of the flux vector $x\in R^{n}$) and the resulting *PP*

$$\{{\left(x_{i},x_{j}\right)}^{T}\in R^{2}\;\;|\;\;x\in P\},$$

that is, the projection of the flux polyhedron P on the $\left(x_{i},x_{j}\right)$-plane. If $x_{i}$ and $x_{j}$ are bounded on P, then the PP is a polytope.

More generally, one may consider ℓ linear objective functions $l_{1},\ldots ,l_{\ell }:R^{n}\to R$,

$$l_{1}(x)=c_{1}^{T}x,\ldots ,l_{\ell }(x)=c_{\ell }^{T}x,$$

and the resulting *objective space*

$$\{{\left(l_{1}(x),\ldots ,l_{\ell }(x)\right)}^{T}\in R^{\ell }\;|\;x\in P\}.$$

(In case ℓ=2 and $l_{1}(x)=x_{i}$, $l_{2}(x)=x_{j}$, the objective space equals the PP defined above). If all objectives are bounded on P, then they vanish on the recession cone of P, and the objective space is a polytope.

Analogously, one may consider ℓ yields $Y_{1},\ldots ,Y_{\ell }:D\to R$,

$$Y_{1}(x)=\frac{c_{1}^{T}x}{d^{T}x},\ldots ,Y_{\ell }(x)=\frac{c_{\ell }^{T}x}{d^{T}x},$$

having different numerators, but the same denominator. They define the yield vector

$$Y(x)={\left(Y_{1}(x),\ldots ,Y_{\ell }(x)\right)}^{T}$$

and the resulting YS

$$\{Y(x)\in R^{\ell }\;|\;x\in P_{>}\}.$$

First of all, the YS is a convex set. Indeed, let $x,y\in P_{>}$ and λ∈[0,1]. By Property 2 (or Proposition 1 in the Appendix),

Y((1−λ)x+λy)=(1−λ′)Y(x)+λ′Y(y),

with λ′∈[0,1], since λ′ depends on *d* (which is identical for all yields), but not on $c_{1},\ldots ,c_{\ell }$, and the map λ↦λ′ is bijective.

By Lemma 3 in the Appendix (extended to yield vectors), if all yields are bounded on $P_{>}$, then the YS is contained in the polytope generated by the yield vectors of generators of $P_{\geq }$ (with defined yield). It can be shown that the closure of the YS equals this polytope. If moreover all yields are attained on $P_{>}$, as in most realistic applications, then the YS equals this polytope.

It remains to specify generators of PPs and YSs. Clearly, a PP, that is, the projection of a flux polyhedron P, is given by all convex combinations of projections of generators of P. Similarly, a YS, that is, the set of yield vectors on the domain $P_{>}$, is given by all convex combinations of yield vectors of generators of $P_{\geq }$.

### 4.3 EFMs and EFVs in phase planes and yield spaces

As just discussed, generators of the flux polyhedron P (or the polyhedron $P_{\geq }$) can be used to generate the PP and the YS. Due to their special properties, we suggest to use EFVs (or EFMs in the special case of a flux cone) as generators. The PP then results as the convex hull of the projected EFVs. The PP can be bounded (if the projected rates are bounded), see Fig. 3(c) and (e), or unbounded, see Fig. 3(a). The YS arises as the convex hull of the mapped EFVs (or EFMs) with defined yield. Yields of interest typically refer to ratios of product(s) excreted *vs.* substrates(s) taken up. In realistic applications, the YS is finite even if fluxes are unbounded, cf. Figs. 3(a) and (b). If a metabolic model is not formulated properly, the YS may become unbounded.

Since PPs and YSs are convex hulls of projected/mapped EFVs (or EFMs in flux cones), all vertices of a PP or a YS correspond to EFVs (or EFMs, respectively), see Fig. 3(a)–(f). In the YS of scenario S1, all EFMs lie on the boundary of the YS, *cf.* Fig. 3(b), while EFMs of more complex and realistic networks may also lie in the interior of the YS. The point (0,0) represents EFM1, a mode that converts S straight into Q. Again, note that the cycle EFM6 does not have a corresponding point in the YS since it has zero substrate uptake $\left(r_{1}\right)$ and product synthesis $\left(r_{3},r_{4}\right)$ rates and thus undefined yields.

Generally, flux vectors with undefined yield (with zero numerator and denominator in the yield function), including the zero flux vector, cannot be mapped to the YS. The YS becomes unbounded (and thus indicates either ill-posed models or ill-posed yields) only if one yield in the YS is infinite due to the existence of flux vectors with product synthesis (nonzero numerator), but without substrate uptake (zero denominator).

For scenario S2, the EFVs are shown in the PP and the YS in Fig. 3(c) and (d), respectively. In contrast to S1, the PP is now bounded in $r_{4}$ and $r_{3}$. It would be unbounded if one of the rates $r_{7}$, $r_{10}$, or $r_{11}$ was used for projection. While the zero vector (EFV23) and the cycle (EFV22) are not represented in the YS, they are contained in the PP: in fact, they are projected to the point (0,0), as EFM7. In the YS, the point (0,0) corresponds only to EFV7 involving reactions R1, R2, and R8.

### 4.4 Computation of phase planes and yield spaces

As explained above, PPs and YSs can be computed as convex hulls of projected/mapped EFVs (or EFMs). In genome-scale metabolic models (GSMMs), however, an enumeration of all these generating vectors is usually not feasible. Nevertheless, in two or three dimensions, PPs and YSs of GSMMs can readily be obtained. PPs can either be approximated by sampling their boundary, using flux variability analysis, or even be exactly computed, *e.g.*, by the convex hull method (Huynh et al., 1992, Lassez and Lassez, 1990). A pseudo-code for sampling the boundary of a PP is given by Algorithm 1 in Table 2.

**Table 2:** Pseudo codes of sampling algorithms for a two-dimensional PP and a two-dimensional YS. Note that in Algorithm 2 optimization is carried out over the auxiliary polyhedron P′ rather than the flux polyhedron P. Otherwise, the two algorithms are structurally identical.

To the best of our knowledge YSs of GSMMs have never been determined or studied in the literature. In fact, a YS can be computed similarly to a PP, thereby using the LP equivalent to the linear-fractional program (LFP), in particular, the auxiliary polyhedron P′, *cf.* Section 2.2. A pseudo-code for sampling the boundary of a YS is given by Algorithm 2 in Table 2.

A discretization parameter of n=20 is often sufficient to approximate PPs and YSs of GSMMs, usually requiring less than one minute computation time.

### 4.5 Implementation in *CellNetAnalyzer*

Our MATLAB^®^ toolbox *CellNetAnalyzer* (von Kamp et al., 2017, Klamt et al., 2007) supports the maximization of both linear (rate) functions (1) and linear-fractional (yield) functions (4) in GSMMs. Moreover, *CellNetAnalyzer* allows one to study rate- and yield-optimal solution sets by means of EFM and EFV analysis if the computation of these generating vectors is feasible. Finally, *CellNetAnalyzer* supports the computation and visualization of PPs and YSs either exactly *via* EFVs (or EFMs) or, as a recent extension for GSMMs, approximately *via* the sampling algorithms described above.

## 5 Production envelopes and yield spaces in strain design

Phase plane (PP) and yield space (YS) analysis are valuable tools in metabolic engineering and (computational) strain design to study the trade-off between biomass and product synthesis in production organisms. Accordingly, growth rate and product synthesis rate are usually chosen to span the PP. In the context of metabolic engineering, PPs are often called production envelopes (PEs) or trade-off plots (Machado and Herrgard, 2015, Maranas and Zomorrodi, 2016). In the following two sections, we will exclusively use the term PEs for PPs. Likewise, biomass and product yields typically span YSs of microbial cell factories.

Various constraint-based modeling methods have been developed to predict rational metabolic intervention strategies that improve the performance of production strains, see Machado and Herrgard, 2015, Maia et al., 2016 for recent reviews. Most of them rely on the concept of (stoichiometric) coupling of growth with product synthesis (Burgard et al., 2003, Klamt and Mahadevan, 2015, von Kamp and Klamt, 2017). Thereby, constraint-based modeling methods are roughly divided into two groups: biased and unbiased approaches (Lewis et al., 2012, Machado and Herrgard, 2015, Maia et al., 2016).

### 5.1 Biased strain design

Biased methods, such as OptKnock (Burgard et al., 2003), OptReg (Pharkya and Maranas, 2006), OptORF (Kim and Reed, 2010), RobustKnock (Tepper and Shlomi, 2010), and others, rely on the assumption that wild type cells as well as constructed mutant strains optimize their metabolism with respect to a fitness function, usually some kind of growth (rate) optimization. Thus, these methods usually operate on PEs, which they try to (re)shape, *e.g.*, by gene/reaction knockouts, such that a sufficiently high product synthesis rate is achieved at optimal growth. Suboptimal growth states are considered less relevant. For instance, the (wild type) strain under the constraints of scenario S3 depicted in Fig. 3(e) already has such a desired shape. At maximal growth rate $r_{4}=7.5$ we can expect a product yield of 0.25 and a production rate of $r_{3}=2.5$. If this performance is sufficient, no intervention will be required. However, if – for whatever reasons – the strain grows suboptimally with less than the maximal growth rate, then little or even no product may be synthesized.

**Fig. 5:**
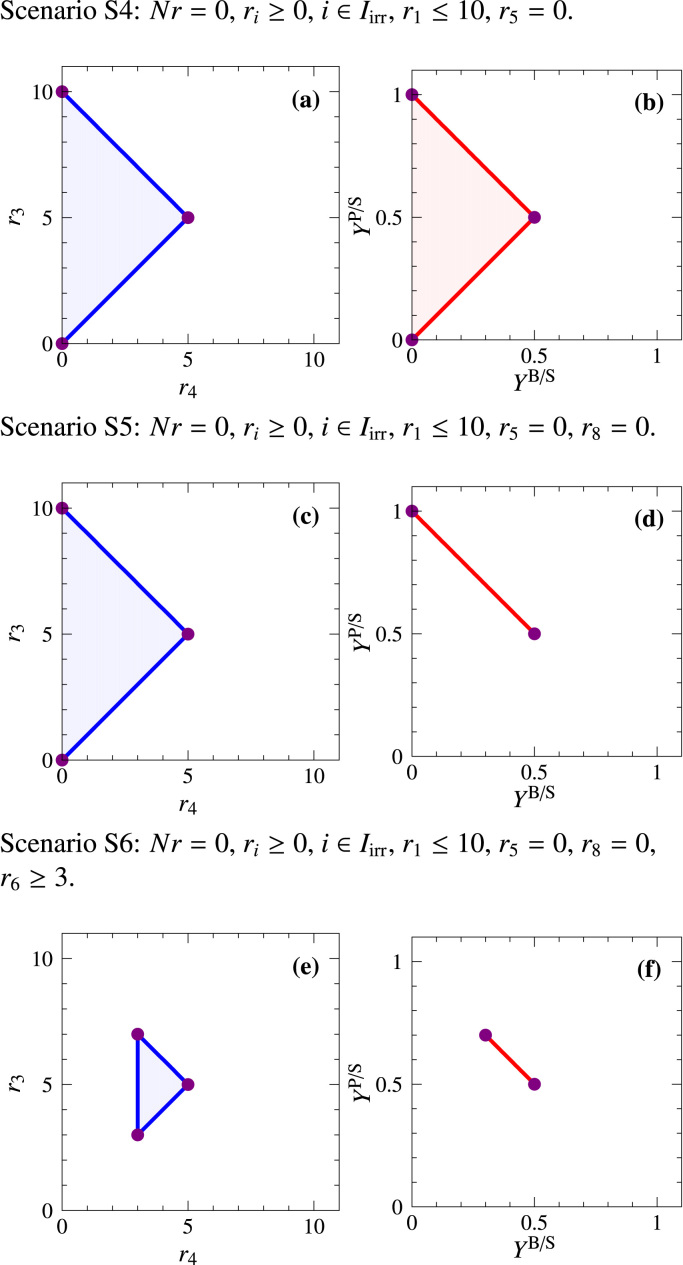
PEs (blue) and YSs (red) for the engineering scenarios S4–S6 of the example network in Fig. 1. The circles correspond to (possibly multiple) EFVs (*cf.*Table 1).

*Scenario S4*. Ideally, the PE of designer strains should contain no solutions on the *x*-axis because then net product synthesis for any nonzero growth rate and thus (strong) growth coupling would be guaranteed. Fig. 5(a) illustrates such a PE for the example network in Fig. 1 (with bounded substrate uptake as in scenario S2) where reaction R5 has been knocked-out. However, flux distributions with low or even zero yields for P and B still exist [*cf.* Fig. 5(b)], namely if the substrate is converted to Q.

### 5.2 Unbiased strain design

Unbiased strain design algorithms, such as minimal metabolic functionalities (Trinh et al., 2008), FluxDesign (Melzer et al., 2009), or minimal cut sets (Klamt and Gilles, 2004, Hädicke and Klamt, 2011, Jungreuthmayer and Zanghellini, 2012), were originally introduced in the context of elementary flux mode (EFM) analysis (for flux cones), restricting their applicability to medium-scale metabolic models without inhomogeneous constraints (flux bounds *etc.)*. While PE analysis is meaningless in unbounded flux cones [see Fig. 3(a)], yields of the respective EFMs are well defined and YS analysis therefore played a central role for unbiased strain design. We illustrate the use of YSs to identify metabolic intervention strategies based on constrained minimal cut sets (MCSs). A constrained MCS is a set of (reaction) knockouts blocking undesired while maintaining desired phenotypes (Hädicke and Klamt, 2011).

*Scenario S5*. In unbiased strain designs, it is often demanded that all EFMs/elementary flux vectors (EFVs) with low product yield are removed from the network. A prototypical YS of this type is illustrated in Fig. 5(d). Such a design guarantees a minimum product yield for every flux vector consuming substrate, in particular, for every flux vector with nonzero growth rate. A suitable MCS that achieves this design is knocking out R5 and R8. Note that although the PEs in Fig. 5(a) and (c) are identical, they represent different states, best illustrated by looking at the solutions projected to (0,0). In both cases the zero vector (EFV23; Table 1) as well as the cycle EFV22 (R7, R10, and R11) are projected to (0,0). However, in the design of Fig. 5(a) it also includes vectors that convert all available S to Q (EFV7 in Table 1). Such fluxes are infeasible in Fig. 5(c). In that way the second design guarantees strong coupling, *i.e.*, high product yields for every flux distribution in the mutant. In fact, it was recently shown that such a growth-coupled overproduction is, in principle, feasible for almost all metabolites in five major host organisms (von Kamp and Klamt, 2017). Hence, the design principle aiming at strong yield coupling of growth and product synthesis has wide applicability.

The computational difficulties associated with MCS analysis (*i.e.*, the restriction to flux cones without inhomogeneous constraints and to medium-scale metabolic models) were resolved in recent years. First of all, by generalizing EFMs to EFVs (Klamt et al., 2017), MCSs can now be computed from the set of EFVs of general flux polyhedra with the same established algorithms developed for the computation of MCSs from EFMs of flux cones (Jungreuthmayer et al., 2013, Hädicke and Klamt, 2011). Furthermore, by transforming a metabolic network to its so-called dual network, a preceding computation of EFMs or EFVs is not needed anymore, and it is now possible to directly compute the smallest MCSs subject to arbitrary linear constraints even in genome-scale networks (von Kamp and Klamt, 2014). Thus, current MCS methods allow to specify both yield and rate constraints in order to obtain the favored shape of the PE and YS of the desired strains. Due to its generality, the method of MCSs can also be employed to find biased intervention strategies as in scenario S4 [Fig. 5(a) and (b)].

Although strong growth coupling can often be achieved in the YS, neither biased nor unbiased strain design methods can be used to find knockout strategies that *guarantee* high synthesis *rates* of the target product. In fact, if the zero flux vector is part of the wild-type solution set, it cannot be removed by just knocking out reactions since deleting reactions cannot enforce a minimum substrate uptake (and thus not a nonzero production rate). However, a highly desirable PE as in Fig. 5(e) would be possible if we were able to upregulate certain fluxes, for example, by overexpressing the associated enzymes. Mathematically, this translates to introducing positive lower bounds for the absolute magnitude of these fluxes.

*Scenario S6*. Enforcing an additional lower bound of $r_{6}\geq 3$ for the strain of scenario S5 will assure a minimum flux of $r_{3}\geq 3$ for P(roduct) formation and thus lead to a PE as in Fig. 5(e).

Computational strain design methods that allow one to predict targeted upregulation of certain fluxes have already been proposed (Mahadevan et al., 2015, Jungreuthmayer and Zanghellini, 2012, Ranganathan et al., 2010, Pharkya and Maranas, 2006). However, experimental implementation of such strains with guaranteed upregulated fluxes is usually much more difficult (if not impossible) than “just” deleting genes or reactions.

## 6 Examples of production envelopes and yield spaces in *E. coli* and their use for strain design

In the following we use a core and a genome-scale metabolic model (GSMM) of *E. coli* to illustrate the value of production envelopes (PEs) and yield spaces (YSs) in analyzing and designing metabolic networks.

### 6.1 Acetate production in *E. coli*

We use EColiCore2 (ECC2) (Hädicke and Klamt, 2017), a recently published core network of the central metabolism in *E. coli*, which reproduces key properties of its genome-scale parent model *i*JO1366 (Orth et al., 2011).

**Fig. 6:**
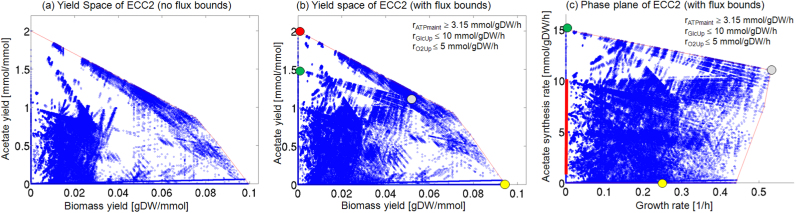
YSs and PE for the production of acetate in *E. coli*, computed for the metabolic core model EColiCore2. (a) YS for acetate and biomass for the flux cone (*i.e.*, without flux bounds), computed by mapping the EFMs of the flux cone (shown as blue dots). (b) YS and (c) PE for acetate and biomass for the flux polyhedron with flux bounds for substrate uptake $\left(r_{\mathrm{GlcUp}}\right)$, non-growth associated maintenance ATP demand $\left(r_{\mathrm{ATPmaint}}\right)$, and oxygen uptake $\left(r_{O2\mathrm{Up}}\right)$. YS (b) and PE (c) were computed by projecting the EFVs of the flux polyhedron (shown as blue dots). In (b) and (c), colors indicate the location of optimal flux vectors; red: maximal acetate yield; yellow: maximal biomass yield; green: maximal acetate synthesis rate; gray: maximal growth rate.

As an example, we analyze the trade-off between biomass and acetate production in ECC2 for growth on glucose (glc) (standard scenario). Initially, we consider the biomass-acetate YS for the flux cone, (without any inhomogeneous constraints). ECC2 is small enough, and we can compute the 558,647 elementary flux modes (EFMs) of its flux cone and map their specific biomass and acetate yields onto the YS as shown in Fig. 6(a). The maximal acetate yield is 2 mmol/(mmol glc) and the maximal biomass yield is close to 0.1 gDW/(mmol glc). Clearly, maximal acetate or maximal biomass yields imply zero production of the other. A PE analysis is not possible as the flux cone is unbounded.

For a more realistic scenario, we introduce a maximal glucose uptake rate of 10 mmol/gDW/h and a non-growth associated, maintenance demand of adenosine triphosphate (ATP) of at least 3.15 mmol/gDW/h. In addition to these standard flux bounds, we assume an oxygen-limited culture with a maximal oxygen uptake rate of 5 mmol/gDW/h. With these three bounds, the flux cone turns into a flux polyhedron, and growth and acetate synthesis rates are now bounded. The resulting flux polyhedron is characterized by 904,599 elementary flux vectors (EFVs), and we can now analyze the YS [Fig. 6(b)] as well as the PE [Fig. 6(c)].

The YS of the flux polyhedron is very similar to that of the flux cone. However, we notice that the biomass yield (reached by 2 EFVs) gets slightly reduced to 0.0945 gDW/(mmol glc) due to the non-growth associated maintenance demand of ATP, which must be produced from glucose, thus reducing the amount of substrate available for biomass synthesis. In contrast, the maximum yield of acetate (exhibited by 676 EFVs) remains constant at 2 mmol/(mmol glc) because ATP can be produced as side product of acetate synthesis.

Comparing YS and PE of the flux polyhedron [Figs. 6(b) and (c)], we see that their shape is quite different. Growth with maximal rate (2 EFVs) is coupled to the production of some acetate while maximal acetate synthesis (748 EFVs) is only achievable without growth. Mapping the EFVs with maximal yields (biomass: yellow; acetate: red) to the PE and, in the other direction, the EFVs with optimal rates (growth: gray; acetate synthesis: green) to the YS reveals non-intuitive relationships. The maximal acetate yield of 2 mmol/(mmol glc) can be reached for acetate production rates between 0.573 and 10 mmol/gDW/h. The lower bound describes the minimum amount of acetate to be produced to reach the maximum acetate yield while simultaneously forming sufficient amounts of ATP for non-growth associated maintenance. The upper bound is a consequence of the limited availability of oxygen as electron sink. At an acetate production rate of 10 mmol/gDW/h, the maximum amount of oxygen has been utilized, and higher acetate production rates require the simultaneous production of fermentation products in order to balance redox, which reduces the acetate yield by 50%. For this reason, flux vectors with rate-optimal acetate synthesis (15 mmol/gDW/h, bounded by the maximum substrate uptake rate) have a reduced yield of 1.5 mmol/(mmol glc) and are thus not yield-optimal.

The situation is similar, but not fully analogous for biomass as product. There are two biomass-yield-optimal EFVs. They use the maximum amount of oxygen available, but only a fraction (*i.e.*, 2.605 mmol/gDW/h) of the possible maximum substrate uptake rate. For larger substrate uptake rates, fermentative pathways with lower biomass yields would have to be used. In contrast to acetate, there is exactly one growth rate (0.246 h^−1^) at which this maximal biomass yield can be reached. For lower growth rates, the relative proportion of the substrate to be used for ATP synthesis for non-growth associated maintenance is larger, thus resulting in lower biomass yields. Finally, the two EFVs with maximal growth rate (0.530 h^−1^) are located in interior of the YS; they are not lying on the boundary, *i.e.*, there are flux vectors with the same biomass yield and higher acetate yield (but the respective rates are lower in these flux vectors).

### 6.2 Ethanol production in a *E. coli* genome-scale model

As mentioned before, the analysis of PEs and YSs is also feasible in GSMMs, where an enumeration of EFMs and EFVs is usually computationally impracticable. In the Supplementary material, Text S1, an example is presented, where we study the trade-off between biomass and ethanol production in the *E. coli* GSMM *i*JO1366 (Orth et al., 2011). We use *CellNetAnalyzer* to compute the biomass-ethanol YS and PE (see Fig. S3) *via* the approximative algorithms given in Section 4.4. In Text S1, we also show a scenario where the maximal specific product synthesis rate can be reached only with maximal product yield and maximal growth rate only with maximal biomass yield (which was not the case in the acetate example discussed above).

### 6.3 Designing *E. coli* acetate producer strains

If we now aim to design an *E. coli* strain for acetate production (with the same core model and flux bounds as used in Fig. 6(b) and (c)) we may apply (biased and unbiased) strain design strategies which differ in the specifications of undesired and protected regions in the PE and/or YS.

*Design D1*. In the sense of a biased strain design, we may demand a minimum acetate production rate of 10 mmol/gDW/h if the cell grows with maximal growth rate. In fact, the PE (Fig. 6(c)) shows that, given the environmental constraints of low oxygen availability, this is already fulfilled in the wild type.

**Fig. 7:**
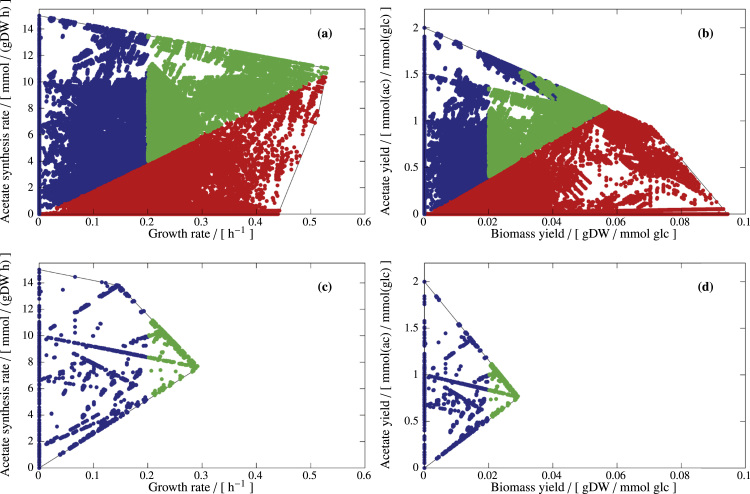
Designing PE and YS for the production of acetate in *E. coli*, according to design strategy D2. Green dots represent desired EFVs (at least one of which must be kept), red dots represent target EFVs (all of which must deleted), and blue dots represent neutral EFVs (which do not interfere with the design objective and therefore may or may not be present in the final design). (a) and (b) specify the desired phenotype, whereas (c) and (d) show the actual structure of the PE and the YS of an exemplary quintuple mutant. In all panels, thin black lines represent the boundaries of the PEs and YSs.

*Design D2*. However, we might fear that the maximum growth rate is not reached by the strain which could then lead to lower or even zero production rates of acetate. We could therefore demand a stronger coupling in the sense that the cell must produce acetate whenever it grows, hence, we search for interventions enforcing a PE similar as in Fig. 5(a). To achieve this we draw a line in the PE that starts at (0,0) and has a certain slope, *i.e.*, a certain ratio (yield) of acetate excretion *vs.* growth rate, see Fig. 7(a) and (b). We may chose a slope of 20 mmol acetate/gDW; the solutions with maximum growth rate are then close to, but still above this line. We now specify all solutions below this line as undesired metabolic behaviors (that must be eliminated); further, all solutions that are above this line *and* have a minimum growth rate of 0.2 h^−1^ as desired phenotypes (of which at least one must be kept). See Fig. 7(a) and (b); individual constraints are illustrated in Fig. S2. Based on this partitioning, we can compute the minimal cut set (MCS), Fig. 7(c) and (d), and observe that the minimum number of reaction knockouts is three. However, with these MCS, a low or even zero acetate yield would still be possible if the cell does not grow, and a low nonzero acetate yield for small growth rates, see example MCS in Fig. 7(c) and (d).

**Fig. 8:**
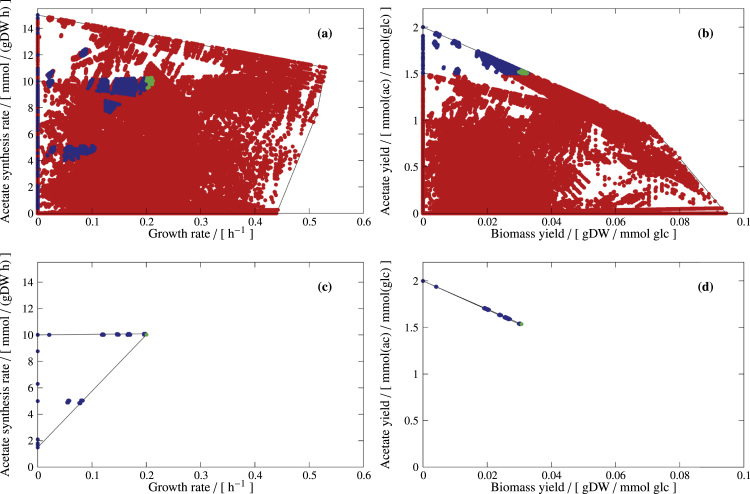
Designing PE and YS for the production of acetate in *E. coli*, according to design strategy D3. Green dots represent desired EFVs (at least one of which must be kept), red dots represent target EFVs (all of which must deleted), and blue dots represent neutral EFVs (which do not interfere with the design objective and therefore may or may not be present in the final design). (a) and (b) specify the desired phenotype, whereas (c) and (d) show the actual structure of the PE and the YS of an exemplary quintuple mutant. In all panels, thin black lines represent the boundaries of the PEs and YSs.

*Design D3*. In an unbiased design approach, we therefore demand a minimum acetate yield of 1.5 mmol/(mmol glc) and again a minimum growth rate of 0.2 h^−1^. See Fig. 8(a) and (b); individual constraints are illustrated in Fig. S2. Thus, for separating desired and undesired phenotypes, we set a horizontal line in the YS and a vertical line in the PE. This combination of design constraints leads to MCSs with a minimum number of seven reaction knockouts, now guaranteeing a high acetate yield, see Fig. 8(c) and (d). Generally, coupling growth with high product *yields* usually requires more interventions than demanding only coupling growth and product synthesis *rates* in the PE.

## 7 Conclusions

Rates and yields of biomass or/and product synthesis are fundamental performance indicators of biotransformation processes. While flux-balance analysis (FBA) provides an established theoretical tool to analyze and predict (optimal) metabolic rates (Lewis et al., 2012) and to design microbial cell factories for optimal (specific) productivity (Maia et al., 2016), similar methods and a rigorous mathematical framework were so far missing for the analysis of optimal metabolic yields. In fact, in the context of FBA, rate and yield optimization were often considered equivalent and FBA, *i.e.* the maximization of rates, was frequently used to also compute yield-optimal solutions. In flux cones, that is, in models where all fluxes are unbounded, setting the substrate uptake rate to a fixed (non-zero) value and then maximizing the product synthesis rate indeed leads to a yield-optimal solution (Schuster et al., 2008, Santos et al., 2011). Equivalent to this FBA-based approach, elementary flux modes (EFMs) have often been used to identify metabolic pathways with optimal yields in flux cones (Prauße et al., 2016). However, in general, the situation is more complex. In applications, constraint-based models typically do contain inhomogeneous (non-zero) flux bounds (Oberhardt et al., 2009), for example, substrate (and/or oxygen) uptake rates within a certain (bounded) range, a minimum ATP maintenance demand rate, *etc.*, which change the solution set from a flux cone to a flux polyhedron. As we unambiguously showed (for our example network, scenario S3, and for acetate synthesis in *E. coli* under oxygen-limited conditions), FBA cannot be used for finding yield-optimal solutions, in general. FBA *always* identifies rate-optimal solutions, which *sometimes* (*e.g.*, when fixing the substrate uptake rate to a specific value), but not always, coincide with yield-optimal solutions.

For the general case, we derived several theoretical results that establish a framework for yield analysis and yield optimization in constraint-based metabolic models:

1. Rather than an ordinary linear program (LP), yield optimization in metabolic networks requires the solution of a linear-fractional program (LFP) for a correct mathematical treatment. Since an LFP can be converted into an LP, yield optimization can efficiently be performed even for genome-scale metabolic models (GSMMs).
2. Production envelopes (PEs) and yield spaces (YSs) are invaluable tools for the rational design of optimal cell factories in metabolic engineering, although they have been confused at times. Indeed, PEs and YSs *sometimes*, but not always, have similar shapes although they carry different information. Moreover, we demonstrated that also YSs can readily be computed in GSMMs.
3. For characterizing yield-optimal solution sets and yield spaces in metabolic networks, elementary flux vectors (EFVs) (or EFMs in case of a flux cone) are extremely useful. It was already known that the set of rate-optimal solutions is spanned by the rate-optimal EFVs (Kelk et al., 2012, Klamt et al., 2017). Similarly, we showed that yield-optimal solutions are convex/conical sums of the yield-optimal EFVs and of EFVs that neither take up substrate nor excrete the product (EFVs with undefined yield). These observations reinforce the fundamental importance of EFVs (or EFMs) as the “coordinates of metabolism” in constraint-based modeling (Zanghellini et al., 2013). Despite the fact that EFMs/EFVs cannot be computed in GSMMs, it is important to understand how they shape yield-optimal and rate-optimal solution sets in metabolic networks.

The methods and algorithms developed are available in our MATLAB toolbox *CellNetAnalyzer* and add an essential building block for constraint-based metabolic modeling and computational strain design.

## Appendix: Mathematical results and proofs

The yield function

$$Y(x)=\frac{c^{T}x}{d^{T}x}$$

is not linear, not even convex (or concave). Still, it is convex in the following sense: Proposition 1 *Let* x,y∈D *and* λ∈[0,1]. *Then for*

z=(1−λ)x+λy∈D

*on the line segment between x and y*, *the yield amounts to*

Y(z)=(1−λ′)Y(x)+λ′Y(y)

*with*

$$\lambda \prime =\frac{\lambda \;d^{T}y}{(1-\lambda )\;d^{T}x+\lambda \;d^{T}y}\in [0,1].$$

*Moreover*, *the map* λ↦λ′ *is bijective. In particular*,

λ′=0ifandonlyifλ=0.

Proof

$$\begin{array}{ccc} Y(z) & = & \frac{c^{T}z}{d^{T}z}\\ & = & \frac{(1-\lambda )\;c^{T}x+\lambda \;c^{T}y}{(1-\lambda )\;d^{T}x+\lambda \;d^{T}y}\\ & = & \frac{(1-\lambda )\;d^{T}x}{(1-\lambda )\;d^{T}x+\lambda \;d^{T}y}\;\frac{c^{T}x}{d^{T}x}\\ & & +\frac{\lambda \;d^{T}y}{(1-\lambda )\;d^{T}x+\lambda \;d^{T}y}\;\frac{c^{T}y}{d^{T}y}\\ & = & \left(1-\lambda \prime )Y(x)+\lambda \prime Y(y\right) \end{array}$$

and

$$\frac{d\lambda \prime }{d\lambda }=\frac{d^{T}x\;d^{T}y}{({\left(1-\lambda )\;d^{T}x+\lambda \;d^{T}y\right)}^{2}}>0.$$

▫

The definition of the yield directly implies the following two results. On the one hand, if there is a vector in $P_{\geq }$ with unbounded yield, then *Y* is unbounded on $P_{>}$. Lemma 1 *Let* $x\in P_{>}$, $y\in P_{\pm /0}$, *and* λ∈[0,1). *Then for*

$$z=(1-\lambda )x+\lambda y\in P_{>}$$

*on the half*-*open line segment between x and y*, *the yield amounts to*

$$Y(z)=Y(x)+\frac{\lambda }{1-\lambda }\frac{c^{T}y}{d^{T}x}.$$

*In particular*,

$$\underset{\lambda \to 1}{\lim\;}Y(z)=\pm \infty .$$

On the other hand, the addition of a vector in $P_{\geq }$ with undefined yield to a vector in $P_{>}$ does not change the yield. Lemma 2 *Let* $x\in P_{>}$ *and* $y\in P_{0/0}$. *Then*,

Y(x+y)=Y(x).

As a consequence, if *Y* is bounded on $P_{>}$, then generators of $P_{\geq }$ cannot have unbounded yield, but may have undefined yield.

In fact, if *Y* is bounded, then the yield of a vector in $P_{>}$ is a convex sum of the yields of generators of $P_{\geq }$ with defined yield. Lemma 3 *Let* $x\in P_{>}$ *be written as* in (18), *that is*,

$$x=\underset{i\in I}{\sum }\alpha_{i}v^{i}+\underset{j\in J}{\sum }\beta_{j}u^{j}$$

*with* $\sum_{i\in I}\alpha_{i}=1$. *If Y is bounded on* $P_{>}$, *then*

$$Y(x)=\underset{i\in I^{d}}{\sum }{\alpha \prime }_{i}Y(v^{i})+\underset{j\in J^{d}}{\sum }{\beta \prime }_{j}Y(u^{j})$$

*with* $I^{d}$ *and* $J^{d}$ *as* in (20),

$${\alpha \prime }_{i},{\beta \prime }_{j}\geq 0,\;\mathrm{and}\;\underset{i\in I^{d}}{\sum }{\alpha \prime }_{i}+\underset{j\in J^{d}}{\sum }{\beta \prime }_{j}=1.$$

*In particular*,

$$\begin{array}{cc} & {\alpha \prime }_{i}=0\;\mathrm{if}\;\mathrm{and}\;\mathrm{only}\;\mathrm{if}\;\alpha_{i}=0\text{,}\\ & {\beta \prime }_{j}=0\;\mathrm{if}\;\mathrm{and}\;\mathrm{only}\;\mathrm{if}\;\beta_{j}=0, \end{array}$$

*and*

$$\underset{i\in I^{d}}{\sum }{\alpha \prime }_{i}>0\;\mathrm{if}\;I^{d}=I.$$

Proof By Lemma 1, if *Y* is bounded on $P_{>}$, then generators of $P_{\geq }$ cannot have unbounded yield. By Lemma 2, the addition of vectors with undefined yield does not change the yield. Hence

$$Y(x)=Y(x^{d})$$

with

$$x^{d}=\underset{i\in I^{d}}{\sum }\alpha_{i}v^{i}+\underset{j\in J^{d}}{\sum }\beta_{j}u^{j}\in P_{>}.$$

Now, consider

$$x\prime =\lambda x^{d}=\underset{i\in I^{d}}{\sum }(\lambda \alpha_{i})\;v^{i}+\underset{j\in J^{d}}{\sum }(\lambda \beta_{j})\;u^{j}\in D$$

with

$$\lambda =\frac{1}{\underset{i\in I^{d}}{\sum }\alpha_{i}+\underset{j\in J^{d}}{\sum }\beta_{j}}>0$$

and hence

$$\underset{i\in I^{d}}{\sum }\lambda \alpha_{i}+\underset{j\in J^{d}}{\sum }\lambda \beta_{j}=1.$$

Using Eq. (15),

$$Y(x^{d})=Y(x^{\prime }),$$

and using Proposition 1 (inductively),

$$Y(x\prime )=\underset{i\in I^{d}}{\sum }{\alpha \prime }_{i}Y(v^{i})+\underset{j\in J^{d}}{\sum }{\beta \prime }_{j}Y(u^{j})$$

with

$${\alpha \prime }_{i},{\beta \prime }_{j}\in [0,1],\;\underset{i\in I^{d}}{\sum }{\alpha \prime }_{i}+\underset{j\in J^{d}}{\sum }{\beta \prime }_{j}=1,$$

and

$$\begin{array}{c} {\alpha \prime }_{i}=0\;\text{if and only if}\;\alpha_{i}=0\text{,}\\ {\beta \prime }_{j}=0\;\text{if and only if}\;\beta_{j}=0. \end{array}$$

If $I^{d}=I$, then

$$\underset{i\in I^{d}}{\sum }\alpha_{i}=\underset{i\in I}{\sum }\alpha_{i}=1$$

and hence

$$\underset{i\in I^{d}}{\sum }{\alpha \prime }_{i}>0.$$

▫ Proof of Theorem 1 Let $x\in P_{>}$ be written as in (18), that is,

$$x=\underset{i\in I}{\sum }\alpha_{i}v^{i}+\underset{j\in J}{\sum }\beta_{j}u^{j}$$

with $\sum_{i\in I}\alpha_{i}=1$. By Lemma 3, the yield of $x\in P_{>}$ is a convex sum of the yields of generators with defined yield, that is,

$$Y(x)=\underset{i\in I^{d}}{\sum }{\alpha \prime }_{i}Y(v^{i})+\underset{j\in J^{d}}{\sum }{\beta \prime }_{j}Y(u^{j})$$

with

$${\alpha \prime }_{i},{\beta \prime }_{j}\in [0,1]\;\;and\;\underset{i\in I^{d}}{\sum }{\alpha \prime }_{i}+\underset{j\in J^{d}}{\sum }{\beta \prime }_{j}=1.$$

If

$$I^{d}=I\,\;\mathrm{and}\;\underset{i\in I^{d}}{\max\;}Y(v^{i})<\underset{j\in J^{d}}{\max\;}Y(u^{j})=:Y^{*},$$

then

$$\underset{i\in I^{d}}{\sum }{\alpha \prime }_{i}>0\;\text{and hence}\;Y(x)<Y^{*}$$

for all $x\in P_{>}$. Now, consider

$$x=v+\beta_{j}u^{j}\in P_{>}$$

with $v\in P_{>}$ and $Y(u^{j})=Y^{*}$ and

$$x\prime =\frac{x}{1+\beta_{j}}=(1-\lambda )v+\lambda u^{j}\in D$$

with

$$\lambda =\frac{\beta_{j}}{1+\beta_{j}}\in [0,1).$$

Using Eq. (15),

Y(x′)=Y(x),

and using Proposition 1,

$$Y(x\prime )=(1-\lambda \prime )Y(v)+\lambda \prime Y(u^{j}),$$

where λ′=1 if and only if λ=1. Hence, $\beta_{j}\to \infty$ implies λ→1, λ′→1, and

$$Y(x)=Y(x\prime )\to Y(u^{j})=Y^{*}.$$

That is, $Y^{*}$ is the supremum. Conversely, if $I^{d}\neq I$ or $\max_{i\in I^{d}}Y(v^{i})\geq \max_{j\in J^{d}}Y(u^{j})$, then the maximum is attained. ▫ Proof of Theorem 2 By Lemma 3, the yield of $x\in P_{>}$ is a convex sum of the yields of generators of $P_{\geq }$ with defined yield. Since the maximum yield is attained, generators with defined, but not maximum yield do not contribute to the optimal solution. Hence, every optimal solution is a convex/conical sum of generators with maximum or undefined yield – and *vice versa*. Thereby, at least one generator with maximum yield contributes to the sum. ▫

## Supplementary material

Application 1
