# Supplementary material for: A mathematical framework for yield (vs. rate) optimization in constraint-based modeling and applications in metabolic engineering
Source: Metab Eng. 2018 May;47:153–69. doi: 10.1016/j.ymben.2018.02.001 (PMC5992331; doi:10.1016/j.ymben.2018.02.001)
Supplement: Application 1 [file mmc1.pdf]

Supplementary material to

A mathematical framework for yield (versus rate) optimization in constraint-based modeling and applications in metabolic engineering

Steffen Klamt[a], Stefan Müller[b], Georg Regensburger[c], Jürgen Zanghellini[d,e]

[a]*Max Planck Institute for Dynamics of Complex Technical Systems, Magdeburg, Germany*
[b]*Faculty of Mathematics, University of Vienna, Austria*
[c]*Institute for Algebra, Johannes Kepler University Linz, Austria*
[d]*Department of Biotechnology, University of Natural Resources and Life Sciences, Vienna, Austria*
[e]*Austrian Centre of Industrial Biotechnology, Vienna, Austria*

Metabolic Engineering 2018. DOI:10.1016/j.ymben.2018.02.001 *February 13, 2018*

Given:

- a flux polyhedron P with bounded yield and
- a set of “bounded” EFVs v (generators of the polytope associated to P) and “unbounded” EFVs u (generators of the recession cone of P) with index sets I^*, I^u (for EFVs v with maximum/undefined yield) and J^*, J^u (for EFVs u with maximum/undefined yield).

If the flux polyhedron is

<table>
<tr><td>a cone,</td><td>a polytope,</td><td colspan="2">a general polyhedron,</td></tr>
<tr><td rowspan="2">then the maximum is attained.
An optimal solution is a
• conical sum of EFVs u with maximum or undefined yield,
$$x^* = \sum_{j\in J^*} \beta_j^* u^j + \sum_{j\in J^u} \beta_j u^j ,$$
where $\sum_{j\in J^*} \beta_j^* > 0$.</td><td rowspan="2">then the maximum is attained.
An optimal solution is a
• convex sum of EFVs v with maximum or undefined yield,
$$x^* = \sum_{i\in I^*} \alpha_i^* v^i + \sum_{i\in I^u} \alpha_i v^i ,$$
where $\sum_{i\in I^*} \alpha_i^* + \sum_{i\in I^u} \alpha_i = 1$
and $\sum_{i\in I^*} \alpha_i^* > 0$.</td><td colspan="2">and has at least one “bounded” EFV v with maximum or undefined yield,
Y / N</td></tr>
<tr><td>Y: then the maximum is attained.
An optimal solution is a
• convex sum of EFVs v with maximum or undefined yield
plus a
• conical sum of EFVs u with maximum or undefined yield,
$$x^* = \sum_{i\in I^*} \alpha_i^* v^i + \sum_{i\in I^u} \alpha_i v^i + \sum_{j\in J^*} \beta_j^* u^j + \sum_{j\in J^u} \beta_j u^j ,$$
where $\sum_{i\in I^*} \alpha_i^* + \sum_{i\in I^u} \alpha_i = 1$
and $\sum_{i\in I^*} \alpha_i^* + \sum_{j\in J^*} \beta_j^* > 0$.</td><td>N: then the maximum is not attained.
An optimal solution in the limit is a
• convex sum of EFVs v with defined (but not maximum) yield
plus a
• conical sum of EFVs u with maximum or undefined yield,
$$x^* = \sum_{i\in I^d} \alpha_i v^i + \sum_{j\in J^*} \beta_j^* u^j + \sum_{j\in J^u} \beta_j u^j ,$$
where $\sum_{i\in I^d} \alpha_i = 1$
and $\sum_{j\in J^*} \beta_j^* \to \infty$.</td></tr>
</table>

Figure S1: Decision tree for the characterization of yield-optimal solution sets in terms of elementary flux vectors (EFVs), according to Theorems 1 and 2.

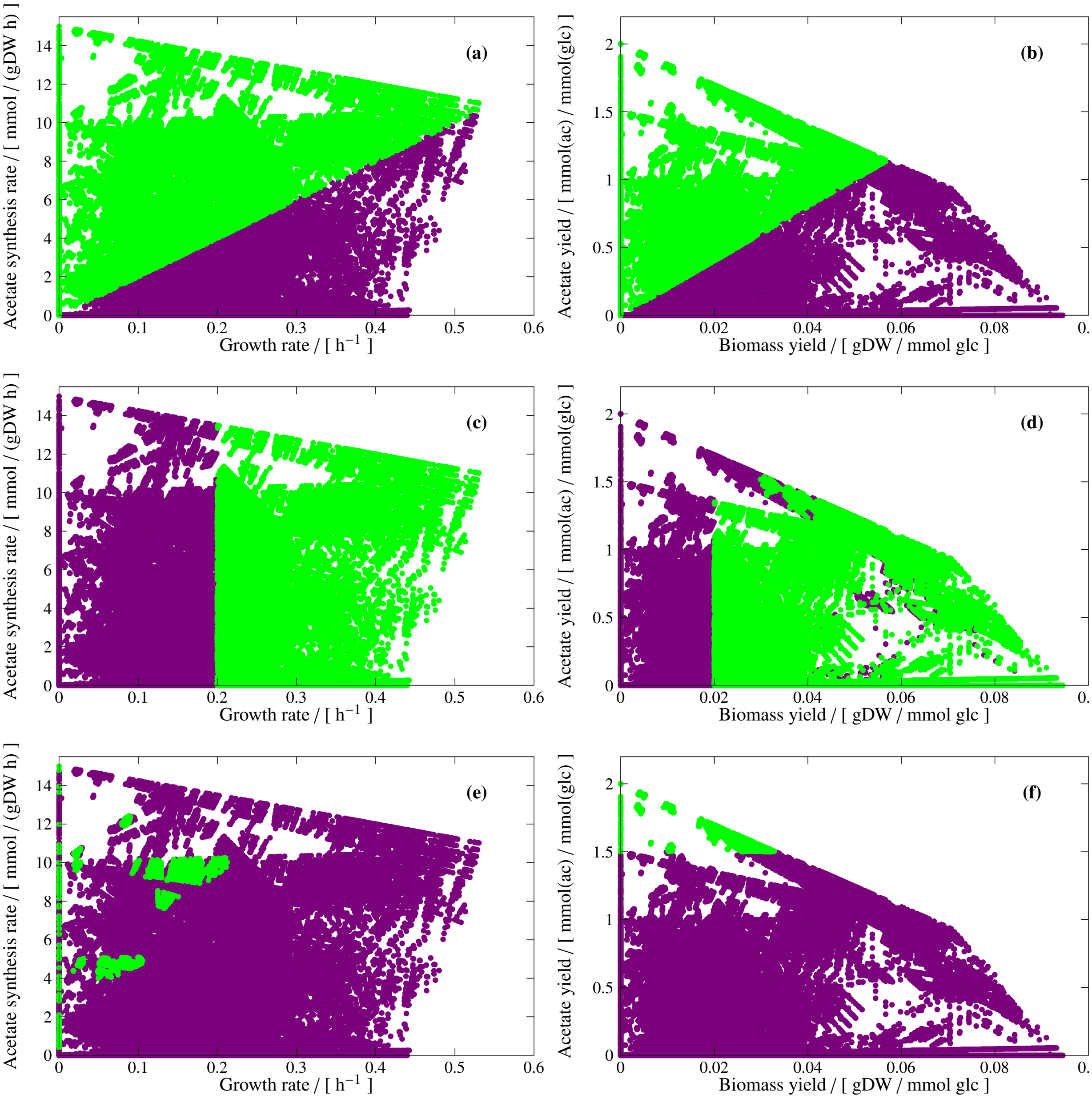

Figure S2: Illustration of the constraints specifying the desired behaviors used in the designs D2 and D3. Panels left and right show the production envelopes (PEs) and yield spaces (YSs), respectively. Panels (a) and (b) illustrate the partition of the feasible fluxes due to a linear minimal acetate secretion as function of growth with a slope of 20 mmol acetate/gram dry weight (gDW); panels (c) and (d) illustrate the partition due to a minimum growth requirement of 0.2 h^{-1}; and panels (e) and (f) illustrate the partition due to a minimum acetate yield requirement of 1.5 mmol/(mmol glucose). Each dot represents (the projection and mapping of) an elementary flux vector (EFV) in the PE and YS, respectively. The intersection of the set of green EFVs in the panels (a) and (c) as well as (b) and (d) gives the set of desired EFVs in the design D2, see Figure 7. Similar, the intersection of the set of green EFVs in the panels (c) and (e) as well as (d) and (f) gives the set of desired EFVs in the design D3, see Figure 8.

Text S1. Production envelope and yield space for ethanol production in a genome-scale model of *E. coli*

To exemplify the analysis of production envelopes (PEs) and yield spaces (YSs) also in a genome-scale metabolic model (GSMM), we analyze the trade-off between biomass and ethanol production in the *E. coli* GSMM model *i*JO1366 [1]. Here, computation of elementary flux modes (EFMs) or elementary flux vectors (EFVs) is not possible and so we use *CellNetAnalyzer* to compute the biomass-ethanol YS and PE via the approximative algorithms given in Section 4.4.

The YS of the flux cone (without additional flux constraints) is shown in Figure S3 (a) and looks similar as for acetate in the core model, see Figure 6(a). The maximal biomass yield is almost exactly the same as in the core model and the maximal ethanol yield is, as expected, 2 mmol/gDW/h.

Next we add the same flux bounds for maximal glucose uptake and for minimal adenosine triphosphate (ATP) non-growth associated maintenance demand as in the acetate example. However, here we assume fully anaerobic growth (with enabled formate-hydrogen lyase (FHL) reaction which was set inactive in the original *i*JO1366 model [1] to reflect aerobic growth). In this case, YS and PE have an identical shape differing only by a scaling factor of ten [the maximal glucose uptake rate; see S3(b) and (c)]. In difference to the acetate example (where oxygen uptake was limited but not zero), the rate-optimal solutions for anaerobic growth and ethanol synthesis correspond to the respective yield-optimal solutions. We see that growth-optimal behavior (with respect to both yield and rate) *may* be accompanied with ethanol synthesis [with an ethanol yield of up to 0.78 mmol/(mmol glucose)]. Yet, ethanol synthesis is not mandatory for maximal growth rate since other pathways with zero ethanol yield are feasible as well. Regarding optimal ethanol yield we find that the maximum yield can be obtained also for smaller ethanol synthesis rates down to 3.15 mmol/gDW/h. This minimal synthesis rate is required to obtain maximum ethanol yield under the constraint of ATP synthesis for non-growth associated maintenance.

Biased and unbiased strain designs (similar as D1-D3 discussed for the acetate example) for growth-coupled ethanol production could now again be computed, even in this GSMM. For example, linear inequalities can be used to specify the undesired and desired regions in the PE and YS which serve as input for the computation of minimal cut set (MCS) via the dual approach presented in [2]. An example for enumerating intervention strategies for growth-coupled ethanol synthesis in a GSMM of *E. coli* can be found in [2] as well.

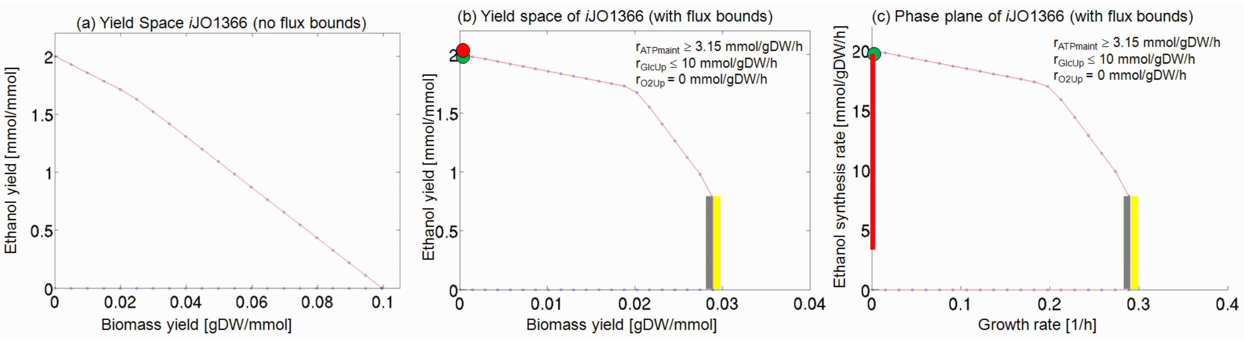

Figure S3: YSs and PEs for the production of ethanol of *E. coli* computed for the GSMM *i*JO1366. (a) YS for ethanol and biomass of the flux cone (*i.e.*, without any flux bounds). (b) YS and (c) PE for ethanol and biomass of the flux polyhedron with flux bounds for substrate uptake (r_{GlcUp}), non-growth associated maintenance ATP demand (r_{ATPmaint}), and zero oxygen uptake (anaerobic growth). In (b) and (c) the colors indicate the location of the following optimal flux vectors: red: maximal ethanol yield; yellow: maximal biomass yield; green: maximal ethanol synthesis rate; gray: maximal growth rate. The red/green circle in (b) as well as the gray/yellow lines in (b) and (c) correspond to exactly the same points/lines and have been slightly shifted for better visibility.

References

[1] J. D. Orth, T. M. Conrad, J. Na, J. A. Lerman, H. Nam, A. M. Feist, B. O. Palsson, A comprehensive genome-scale reconstruction of Escherichia coli metabolism – 2011, Mol Syst Biol 7 (2011).

[2] A. von Kamp, S. Klamt, Enumeration of Smallest Intervention Strategies in Genome-Scale Metabolic Networks, PLoS Computational Biology 10 (2014) e1003378.
